# Study protocol to assess aflatoxin M1 health risks versus benefits of dairy consumption in Ethiopian children: an epidemiological trial and risk-benefit analysis

**DOI:** 10.1136/bmjopen-2024-084257

**Published:** 2024-04-28

**Authors:** Felicia Wu, Derek Headey, Kalle Hirvonen, Ashish Pokharel, Masresha Tessema

**Affiliations:** 1 Department of Food Science and Human Nutrition, Michigan State University, East Lansing, Michigan, USA; 2 Department of Agricultural, Food, and Resource Economics, Michigan State University, East Lansing, Michigan, USA; 3 International Food Policy Research Institute, Washington, District of Columbia, USA; 4 Ethiopian Public Health Institute, Addis Ababa, Ethiopia

**Keywords:** Epidemiologic studies, Community child health, Public health, Nutrition & dietetics

## Abstract

**Introduction:**

In Sidama, Ethiopia, animal-source foods can be difficult to access. Milk has important nutrients for child growth, but carries the risk of aflatoxin M_1_ (AFM_1_) contamination. AFM_1_ is a metabolite of the mycotoxin aflatoxin B_1_ (AFB_1_) in dairy feed; cows secrete AFM_1_ in milk when their feed contains AFB_1_ produced by *Aspergillus* fungi in maize, nuts and oilseeds. It is unknown whether AFM_1_ compromises child growth and health.

**Methods and analysis:**

This protocol paper describes our study in Sidama to determine the impact of milk consumption and AFM_1_ on child growth in the first 18 months of life. We will collect baseline and end-line data on dairy production, socioeconomic and nutritional factors of 1000 dairy-owning households with children ages 6–18 months at baseline; and gather samples of milk and dairy feed and child anthropometrics. We will conduct phone interviews every 6 months to ascertain changes in practices or child health. Dairy feed will be tested for AFB_1_; milk for AFM_1_, pathogens and nutrients. Controlling for herd size, socioeconomic, nutritional and behavioural factors, we will determine the association between child anthropometrics and milk consumption, as well as AFM_1_ exposure. We will examine whether AFM_1_ exposure affects child growth in the first 18 months of life, and weigh the benefits and risks of milk consumption.

**Ethics and dissemination:**

The protocol is approved by the Institutional Review Boards of the Ethiopian Public Health Institute (EPHI-IRB-481–2022), Michigan State University (STUDY00007996) and International Food Policy Research Institute (DSGD-23–0102). Written informed consent will be obtained from all participants, who may withdraw from the study at any time. Confidentiality of collected data will be given high priority during each stage of data handling. The study’s findings will be disseminated through stakeholder workshops, local and international conferences, journal articles and technical reports.

**Strengths and limitations of this study**

- This is the first study that assesses how aflatoxin M_1_ (AFM_1_) in milk affects child growth in Ethiopia, controlling for socioeconomic, nutritional and environmental factors.
- This research compares the health risks of AFM_1_ with the benefits of milk consumption for children.
- The findings will be used to inform feasible, health risk-based policies on AFM_1_ in milk, not just in Ethiopia, but potentially in other nations worldwide.
- The findings of this study would not allow for causal inference or finding a toxicological mechanism because of the observational design, but would aid in the understanding of the relationship between AFM_1_ exposure and child growth.

## Introduction

Aflatoxins are secondary metabolites of the fungi *Asperfillus flavus* and *A. parasiticus*, which frequently infect food and feed crops such as maize, groundnuts, oilseeds and tree nuts.^1^ Aflatoxin B_1_ (AFB_1_) is the most toxic form of aflatoxin, and a potent liver carcinogen. Aflatoxin M_1_ (AFM_1_), a metabolite of AFB_1_, is excreted in milk when mammals consume feed contaminated with AFB_1_. Infants and young children can become exposed to AFM_1_ through dairy animal milk consumption. Although AFM_1_ is a far less potent carcinogen than AFB_1_, it may cause other health risks. Furthermore, knowledge of the tradeoff between the nutritional benefits of milk versus risks of AFM_1_ exposure in milk is currently sparse.

Globally, milk is a critically important animal source of food for infants and young children, providing a wide range of nutrients—protein, carbohydrates, fat, calcium, potassium and vitamin B_12_ among others—crucial for growth and development.^2–4^ Including milk in children’s diets is all the more important when access and affordability of other animal-sourced food such as meat and eggs are difficult.^5^ In addition, milk products are economically valuable; as the dairy value chain contributes substantially to livelihoods in semi-urban and rural areas worldwide.^6–8^

Conversely, milk can harbour multiple contaminants such as AFM_1_. AFB_1_, the parent compound of AFM_1_, may impair child growth^9^ through several pathways such as disruption of insulin-like growth factors,^10^ intestinal damage^11^ and immunosuppressive effects that increase infection susceptibility.^12 13^ Whether AFM_1_ could cause similar effects is unknown. This research study aims to fill this knowledge gap by examining the relationship between AFM_1_ exposure and growth outcomes in infants and young children in Ethiopia.

Ethiopia is an ideal setting for our proposed work, as it faces important tradeoffs between the benefits of milk production and consumption and AFM_1_ risk. Ethiopia has recently adopted a strict standard for AFM_1_ in milk, 0.5 µg/L (Ethiopian Standards Agency CES-278), which is often exceeded in raw milk samples from Ethiopia as shown by mean AFM_1_ levels ranging between 0.41 and 0.69 µg/L with a range of 0.028–4.96 µg/L.^14^ The action level for AFM_1_ in milk in the USA is the same, at 0.5 µg/L^15^; while the European Union standard is 0.05 µg/L.^16^ The recent lack of evidence linking AFM_1_ to cancer has led to the question of whether the standards set are reasonable.^12^ Moreover, dairy markets are important in Ethiopian livelihoods, particularly in rural areas where dairy is an important source of supplemental income.^17^ If excessively strict AFM_1_ standards are enforced in Ethiopian dairy markets, it could result in both economic losses from discarded milk, and nutritional losses due to negative public perception against milk consumption.^12 18^

To assess the relationship between AFM_1_ exposure and growth outcomes and the potential benefits and risks of AFM_1_ in milk in Ethiopia, we use a prospective longitudinal cohort design. Here, we describe the objectives, methods and expected outcomes of this study.

## Objectives

Our goal is to determine whether AFM_1_ exposure through milk consumption is associated with child growth impairment over a period of 18 months in early childhood; then to compare this risk with the benefits of milk consumption. This evidence will inform policy recommendations for AFM_1_ regulation in dairy products in Ethiopia. The specific objectives of this study are to:

1. Determine the association between milk consumption and growth outcomes in children 6–36 months of age.
2. Examine the association between AFM_1_ exposure and growth outcomes in children 6–36 months of age who consume milk.
3. Compare the health benefits and risks associated with dairy consumption in relation to child growth outcomes.
4. Develop policy recommendations for regulating AFM_1_ and communicating dairy benefits and risks.

## Methods and analysis

The study will be carried out in two parts. First, we will conduct formative research with semi-structured interviews of families with young children aged 6–18 months who also own dairy cows, to assess dairy management practices (especially those related to cattle feeds) and dairy production and consumption practices in the Sidama region in Ethiopia. Second, we will use findings to inform the main quantitative arm of the study as outlined in the four objectives above.

### Formative research study

Limited prior evidence suggests that AFM_1_ contamination in milk is widespread in Ethiopia.^19 20^ There is also limited evidence on farmers’ awareness of AFB_1_ contamination in feed and consequently AFM_1_ contamination in milk, feed and storage practices used to reduce contamination (if any), and behaviours of children’s caregivers in relation to these issues. Hence, we conducted a formative research study to gauge the AFM_1_ risk in cow milk in peri-urban and rural study areas with high rates of cattle ownership and variation in market access to feed. Through the formative research, we gained an understanding of cow feeding issues such as feed type, variation and storage; as well as children’s dairy consumption patterns and complementary feeding practices. The findings of the formative research informed the sampling strategy and the questionnaire design of the main study. The results of the formative research are presented in a different research paper.^21^

### Study design and data collection

The target group of the main study is 1000 caregivers and their children aged 6–18 months from urban or peri-urban households that raise dairy cattle. In the initial visit, a baseline survey will be used to collect data such as anthropometric measurements, child’s diet and socio-demographic factors among other data. After the baseline survey, short phone-based interviews will be carried out every 6 months to assess the variability in the child’s milk consumption and dairy production factors. Our end-line visit, mainly to collect data on child’s anthropometric measurements, will be carried out 18 months after the initial visit. **Patient and public involvement**: the interview results from the formative research described above on public participants in Sidama, Ethiopia, aided the selection of households in the second part of our work.

### Study setting

**Figure 1:**
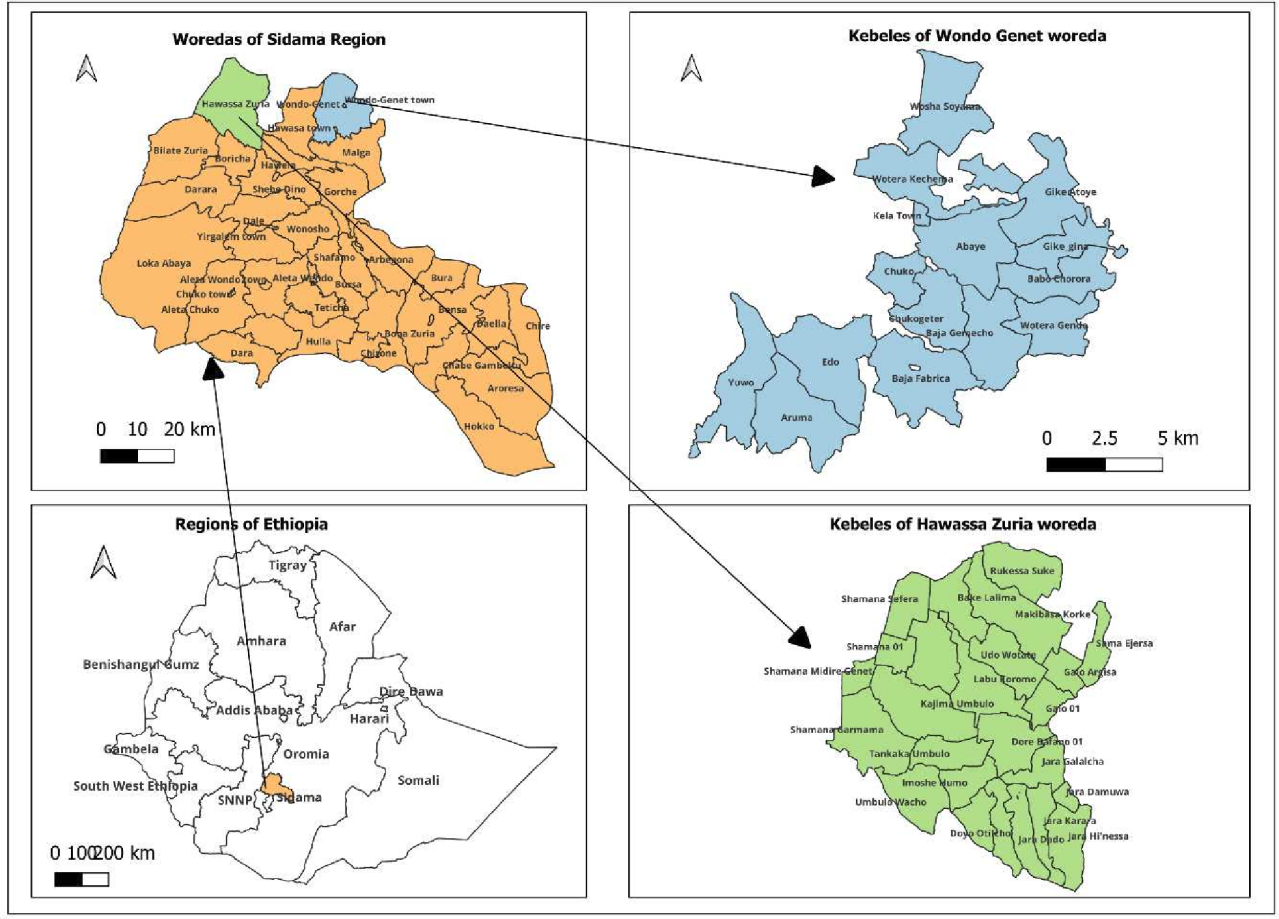
Maps of the study areas for the proposed work in Sidama, Ethiopia.

The study will be conducted in two districts, Wondo Genet and Hawassa Zuria of Sidama region in Ethiopia (figure 1). These districts are purposely selected due to the common practice of using cow’s milk as complementary food for young children, availability of commercial milk, potential for AFB_1_ contamination in livestock feeds and variation in market access to feed.

### Sample size calculations

Using the 2016 Ethiopia Demographic Health Survey^22^ and focusing on our primary anthropometric outcome variable, we have mean height-for-age z-scores (HAZ) in children under the age of 36 months of −1.46 (SD=1.56). With the significance level set at 5%, power at 80%, allowing for 10% attrition between survey rounds, and accounting for that not all children consume milk, the study requires approximately 1000 children to detect a 0.3 SD improvement in child HAZ. This minimum detectable effect size corresponds to the estimated association between household cow ownership and child HAZ (children starting at 6–18 months of age) in a study by Hoddinott *et al* in rural Ethiopia.^23^ Since we focus directly on children’s dairy consumption rather than cattle ownership, and our exposure variable is continuous rather than dichotomous, the expected magnitude of the association is likely to be larger than the association documented in Hoddinott *et al*.^23^ Thus, we expect this sample size to offer sufficient precision to detect positive and/or negative associations between AFM1 exposure and growth outcomes in this population.

### Sampling procedures

Our formative research found that feed ingredients and type varied by herd size and season. Based on these findings, we will classify the dairy cow-owning households into small herd size (one to three cattle) and large herd size (four or more cattle) and randomly select a total of 500 households each from both groups. The two study districts have a list of herd sizes to provide the sampling frame. Within these two groups, all households have an equal chance of being selected.

### Outcome variables

Anthropometric status indicators are the main outcome variables for this study. Anthropometric measurements—weight, height/length, mid-upper arm circumference (MUAC) and head circumference—will be collected from the children at baseline and end line. Height/length will be measured using height/length boards, with the measurements recorded to the nearest 0.1 cm. Weight will be measured using a digital weighing scale (Seca 874 digital floor scale) and recorded to the nearest 0.1 kg. Head circumference will be measured by using a Seca paediatric head circumference measuring band to the nearest 0.1 cm decimal.

Using the WHO 2006 child growth standards, we will calculate HAZ, weight-for-height z-scores (WLZ), weight-for-age z-scores (WAZ) and head circumference-for-age z-scores (HCZ). The HAZ is the primary variable of interest and as secondary outcomes, we will consider the dichotomous variable of stunting (ie, HAZ below −2 SD), WLZ and wasting (ie, WLZ below −2 SD), WAZ and underweight (ie, WAZ below −2 SD), MUAC and HCZ.

### Explanatory variables

Child’s milk consumption and AFM_1_ levels in milk are the key explanatory variables in this study. Child’s milk consumption will be measured through a 1-day quantitative multiple-pass 24-hour recall interview, consisting of four steps designed to enhance respondent’s recall accuracy.^24^ We will ensure that all days of the week are proportionately represented during the dietary recall, to account for day-of-the-week effects on food intake. We will also collect a second, non-consecutive day 24 hours recall (repeat), within 2–10 days of the first recall, from a randomly selected subsample of children to account for the day-to-day variability of dietary intake within individuals and for nutrients estimation.

At the baseline and the end-line visits, we will collect milk samples (2000 samples in two rounds). AFM_1_ testing in milk, analysis of dilution of milk, nutrients composition and pathogens testing (including *Escherichia coli and Campylobacter* spp) will be conducted at Ethiopian Public Health Institute (EPHI) laboratory following standard operating practices for handling, processing and storing samples.

AFM_1_ in milk samples will be detected by using a commercial ELISA kit (catering to catalogue no. 961AFLM01C-ULTRA), manufactured by Helica Biosystems of Santa Ana, California, USA.^25^ The methodology for conducting the test will be based on the manufacturer’s guidelines. Standards and samples of 200 µl will be replicated in precoated plates. The resulting mixture will be incubated for 2 hours, followed by washing and the addition of conjugates. After the completion of 15 min of additional incubation and subsequent washing, enzyme substrate of 100 µl will be added to each well, permitted to incubate for 15 min and afterwards stopped using 100 µl of stop reaction. Finally, optical density measurements will be taken at 450 nm for each microwell using a microplate reader. Calculation of AFM1 levels achieved by means of a logarithmic standard curve will require an R^2^ value of no less than 95%. The average results from duplicate readings will be used for the presentation of results.^25^

### Control variables

Data on the control variables will be collected during the survey visits and will include characteristics that are likely to be correlated with child growth outcomes and child’s milk intake. This includes variables such child’s sex, age and diet, parent’s education, household wealth index, milk’s nutrient content, pathogens in milk and child’s maize consumption.

A questionnaire will collect information about child, parent and household characteristics. Information on children’s diet comes from the 24-hour recall survey. We will control for children’s diets by calculating caloric intake variables from eight food groups: (1) Breastmilk, (2) Grains, roots and tubers, (3) Pulses, nuts and seeds, (4) Dairy products, (5) Flesh foods (meats, fish, poultry, organ meats), (6) Eggs, (7) Vitamin-A rich fruits and vegetables and (8) other fruits and vegetables.^26^ Additionally, we will collect detailed data on the non-standard recipe’s ingredients for all mixed dishes that were prepared at home and use standard recipes from the National Food and Nutrition baseline survey dietary data set to assess the amount of food consumed.^27^ The dietary surveys will be administered outside of major Ethiopian Orthodox fasting periods during which dairy household availability and consumption may decline sharply.^28^

One potential source of measurement error in linking food consumption to child growth is within-person variability in consumption. Since linear growth in young children depends on long-term dietary quantity and quality, the 24-hour recall method is imperfect for capturing variability in diets over time.^29^ To address this, we will conduct interim phone surveys every 6 months to ask caregivers about the 24-hour milk consumption and weekly recall of the child’s diet diversity, and assess basic milk production. These higher frequency measurements will be used to assess seasonality, variability, and to adjust for consumption variability in our analysis.

A subsample of 500 livestock feed samples will be collected for testing AFB_1_. Detection of AFB_1_ in feed samples will be achieved using the Helica Low Matrix Aflatoxin B_1_ competitive ELISA kit, manufactured by Helica Biosystems, Santa Ana, California, USA, listed under Catalogue Number 981BAFL01LM-96 and using procedures outlined by them.^30^

While estimating the child’s actual amount of milk intake, the dilution of milk by water, a common practice in the Ethiopian livestock sector, is a key concern. The level of dilution from water may not be captured accurately through a survey question, as farmers may under-report such information due to social desirability bias. To address this issue, we will test the milk samples for nitrogen content (approximating protein). The total nitrogen content of the milk will be determined by the Kjeldahl method.^31^

The milk samples’ nutrient composition will be obtained from an ongoing analysis of Ethiopian Food Composition tables. However, we will re-analyse randomly selected subsamples to check if their nutrient composition is similar to those reported in the Ethiopian Food Composition table. In brief, proximate composition analysis, moisture content, crude fibre, crude protein and total ash of milk will be determined; with official method numbers 925.09, 2001.11, 4.5.01, 920.169, and 923.03, respectively.^32^ The calcium content of milk will be measured at the EPHI laboratory using an atomic absorption spectrophotometer, following the method no. 985.35 for Ca.^32^ The milk samples will be analysed for pathogens *E. coli* and *Campylobacter* spp. using the general procedures outlined in the Food and Drug Administration’s Bacteriological Analytical Manual BAM^33^ and ISO 10272-1:2017 (*for Campylobacter* spp). We expect that the milk samples found in the study will contain a high number of microbial florae in its background and low numbers of *Campylobacter* spp.

### Study timeline

For the main study, field activities will commence in April 2024 and all data collection activities will be completed by August 2025. Before the start of the quantitative survey, a 1-day stakeholder participatory meeting will be held to inform about the survey goals and seek consent to proceed with the study activities. Data management, monitoring and data analysis will be a continuous process throughout the study period. Results dissemination to the stakeholders will be carried out in December 2025.

### Data quality assurance

15 days of training for field research assistants and supervisors will be provided. After training on methodological procedures, questionnaires and quality assurance, the questionnaires will be tested in a pilot group and adapted based on the received feedback from the study team. The questionnaires will be translated into the local language (Sidamigna) and back-translated to English to ensure the quality of the translation. Supervisors will receive additional training on teamwork and on monitoring and supervising the data collection process. For laboratory analysis, a quality control chart will be used to ensure the internal and external quality control materials are in the acceptable range.

### Data analysis

#### Empirical approach

Descriptive analysis will focus on computing frequencies and percentages for categorical variables and summary statistics (like means, medians SD, IQR) for summarising continuous variables. To determine the association between dairy consumption and growth outcomes in children 6–18 months of age at the baseline, we will specify a least squares regression model as:

(1) $$Y_{iv}=\alpha +\beta_{M}M_{iv}+X_{iv}^{\prime }\delta +\varepsilon_{iv}$$

where $Y_{iv}$ is the anthropometric outcome for a child *i* residing in enumeration area *v* measured at various time points. Variable $M_{iv}$ measures the child’s milk intake at various time points. The regression controls for child, parent and household characteristics that are captured in vector $X_{iv}$ . These control variables are observable characteristics that are likely to be correlated both with a child’s milk intake and his/her growth outcomes. The terms α and $\varepsilon_{iv}$ in equation (1) represent the intercept and error term, respectively. The coefficient $\beta_{M}$ quantifies the conditional association between a child’s milk intake and subsequent growth outcome, measured either as HAZ at the end line or as change in HAZ between the baseline and end-line surveys. Considering previous evidence from Ethiopia and elsewhere, we expect $\beta_{M}$ >0.

To determine the association between AFM_1_ levels in milk and nutrient density in milk, we will conduct a univariate analysis on each of the macronutrients and micronutrients and then carryout bivariate tests with AFM_1_ levels in the same samples and determine if and how they are associated with each other.

To determine the association between AFM_1_ exposure and growth outcomes in children 6–36 months of age who consume dairy, we will test whether the consumption of AFM_1_-contaminated milk is associated with lower HAZ (ie, more growth faltering) controlling for confounders and co-factors, including the amount of milk consumed and nutrient content and pathogens in the milk. We will append equation (1) with a variable capturing the child’s AFM_1_ exposure (variable $A_{iv}$ ). In this model, we also control for the estimated macronutrient and micronutrient content and pathogens (vector $C_{iv}^{`}$ ) in the milk sample collected at the baseline from the household in which the child resides. We will also control for the child’s maize consumption (vector $H_{iv}^{`}$ ), since maize is also likely to be contaminated by aflatoxins in the Ethiopian setting.^34–37^ The estimated model can be defined as:

(2) $$Y_{iv}=\alpha +\beta_{M}M_{iv}+\beta_{A}A_{iv}+\beta_{C}C_{iv}^{\prime }+X_{iv}^{\prime }\delta +H_{iv}^{\prime }\vartheta +\varepsilon_{iv}.$$

We expect that $\beta_{A}$ < 0.

To compare the health benefits versus risks of dairy consumption in child growth outcomes, we will use the regression estimates from equation (2) to conduct joint hypothesis tests for $\beta_{M}$ and $\beta_{A}$ , where the null hypothesis is that both of these coefficients are zero, to inform benefit-risk tradeoff of dairy consumption in the context of AFM_1_.

#### Estimation methods

The primary outcome variables in most of our statistical models will be continuous and will therefore typically be estimated using the Ordinary Least Squares method. As secondary outcomes, we will also examine dichotomous outcomes—such as stunting or wasting—as dependent variables, in which case we will use both linear probability models and Probit models to implement the regression analysis. Homoscedasticity in the residuals will be tested and if rejected, the SEs will be adjusted for heteroscedasticity following.^38^ All statistical analyses will be conducted using Stata, V.17 or higher.

### Ethics and dissemination

The study protocol is approved by the Institutional Review Board of the Ethiopian Public Health Institute (protocol no: EPHI-IRB-481–2022), Michigan State University (protocol no: STUDY00007996) and International Food Policy Research Institute (protocol no: DSGD-23–0102). Written informed consent will be obtained from each respondent and participants will be informed that they may withdraw from the study at any time. Confidentiality of all collected data will be given high priority during each stage of data handling. Individual names and personal information of respondents will be kept confidential and data sets will be kept anonymous for analysis. Patient consent for publication is not required.

The study’s findings will be disseminated through several communications channels, including stakeholder workshops, various local and international conferences and technical reports. Additionally, the findings will be shared through research articles and submitted for publication in peer-reviewed journals.

## Discussion

The results of this study will inform researchers and policymakers on the impact of AFM_1_ in milk on children’s nutrition outcomes and will have implications for the transforming dairy value chains in Ethiopia. Ethiopia has recently adopted strict AFM_1_ standards in milk and other dairy products, which has led to dairy farmers dumping their milk instead of selling it in the market.^12^ An earlier study showed that cancer risk from AFM_1_ consumption was expected to be extremely low.^12^ However, if AFM_1_ has other harmful health effects, such as compromising child growth, then the strict regulations could be justified. In this study, we can determine whether AFM_1_ levels are higher when animals are fed particular feedstuffs, which—if AFM_1_ is correlated with poorer child growth outcomes—will help identify which crops should not be included in dairy feed. But if our work finds that AFM_1_, independently of livestock feed, causes poor child growth outcomes, then the focus need not just be on reducing AFB1 in dairy feed, but also on technologies that can remove AFM_1_ from milk before it is given to children or further processed for other dairy products.

Our work will contribute to rational policymaking and risk communication at local and national levels on whether AFM_1_ poses a risk to children’s health. It will help policymakers and risk communicators in crafting messages balancing the benefits, risks and health outcomes associated with dairy consumption. Secondarily, there could be economic benefits to dairy producers if AFM_1_ is found to have minimal (or no) adverse child growth outcomes, so that standards and regulations can be set at levels appropriate to the true risk, and so that dairy products do not need to be discarded because of overly stringent regulations.^4 39^

There are some limitations of our study. Since a randomised control trial in this context is not feasible, we use a quasi-experimental approach to understand the risks and benefits of child milk consumption in this context and aim to minimise the risk of bias by controlling for observable confounding factors. We use the 24-hour dietary recall and the limitation of this method is that it only captures food intake in the 24 hours prior to the interview. To address this, we will collect data on the consumption of food groups in the past 7 days and also ask whether the milk quantity consumed in the last 24 hours reflects the typical quantity consumed by the child.

Despite these limitations, the results of our study will inform policy decisions on the appropriate AFM_1_ standards for commercial milk and dairy products, on measures to mitigate AFM_1_ risks and on improvement of knowledge of AFM_1_ risks among dairy consumers, producers, and other dairy value chain actors.

## Supplementary Material

Reviewer comments

Author's manuscript
