# Supplementary material for: Study protocol to assess aflatoxin M1 health risks versus benefits of dairy consumption in Ethiopian children: an epidemiological trial and risk-benefit analysis
Source: BMJ Open. 2024 Apr 28;14(4):e084257. doi: 10.1136/bmjopen-2024-084257 (PMC11057243; doi:10.1136/bmjopen-2024-084257)
Supplement: Author's manuscript [file bmjopen-2024-084257.draft_revisions.pdf]

BMJ Open is committed to open peer review. As part of this commitment we make the peer review history of every article we publish publicly available.

When an article is published we post the peer reviewers' comments and the authors' responses online. We also post the versions of the paper that were used during peer review. These are the versions that the peer review comments apply to.

The versions of the paper that follow are the versions that were submitted during the peer review process. They are not the versions of record or the final published versions. They should not be cited or distributed as the published version of this manuscript.

BMJ Open is an open access journal and the full, final, typeset and author-corrected version of record of the manuscript is available on our site with no access controls, subscription charges or pay-per-view fees (http://bmjopen.bmj.com).

If you have any questions on BMJ Open's open peer review process please email info.bmjopen@bmj.com

BMJ Open

Study protocol to assess aflatoxin M1 health risks vs. benefits of dairy consumption in Ethiopian children:An epidemiological trial and risk-benefit analysis

Journal:	*BMJ Open*
Manuscript ID	bmjopen-2024-084257
Article Type:	Protocol
Date Submitted by the Author:	14-Jan-2024
Complete List of Authors:	Wu, Felicia; Michigan State University, Department of Food Science and Human Nutrition; Michigan State University, Department of Agricultural, Food, and Resource Economics<br>Headey, Derek ; IFPRI, Poverty, Health and Nutrition Division<br>Hirvonen, Kalle ; International Food Policy Research Institute<br>Pokharel, Ashish; Michigan State University, Department of Agricultural, Food, and Resource Economics<br>Tessema, Masresha; Ethiopian Public Health Institute,
Keywords:	EPIDEMIOLOGIC STUDIES, Community child health < PAEDIATRICS, PUBLIC HEALTH, NUTRITION & DIETETICS

SCHOLARONE™
Manuscripts

Study protocol to assess aflatoxin M1 health risks vs. benefits of dairy consumption in Ethiopian children: An epidemiological trial and risk-benefit analysis

Felicia Wu[1,2*], Derek Headey[3], Kalle Hirvonen[3], Ashish Pokharel[2], Masresha Tessema[4]

[1] Department of Food Science and Human Nutrition, Michigan State University, East Lansing, MI, USA

[2] Department of Agricultural, Food, and Resource Economics, Michigan State University, East Lansing, MI, USA

[3] International Food Policy Research Institute

[4] Food Science and Nutrition Research Directorate, Ethiopian Public Health Institute, Addis Ababa, Ethiopia

* Correspondence to Dr. Felicia Wu, fwu@msu.edu

For peer review only

ABSTRACT

Introduction. In Sidama, Ethiopia, animal source foods can be difficult to access. Milk has important nutrients for child growth, but carries the risk of aflatoxin M1 (AFM1) contamination. AFM1 is a metabolite of the mycotoxin aflatoxin B1 (AFB1) in dairy feed; cows secrete AFM1 in milk when their feed contains AFB1 produced by *Aspergillus* fungi in maize, nuts, and oilseeds. It is unknown whether AFM1 compromises child growth and health.

Methods and analysis. This protocol paper describes our study in Sidama to determine the impact of milk consumption and aflatoxin M1 on child growth in the first 18 months of life. We will collect baseline and endline data on dairy production, socioeconomic, and nutritional factors of 1000 dairy-owning households with children ages 6-18 months at baseline; and gather samples of milk and dairy feed and child anthropometrics. We will conduct phone interviews every six months to ascertain changes in practices or child health. Dairy feed will be tested for AFB1; milk for AFM1, pathogens, and nutrients. Controlling for herd size, socioeconomic, nutritional, and behavioral factors, we will determine the association between child anthropometrics and milk consumption, as well as AFM1 exposure. We will examine whether AFM1 exposure affects child growth in the first 18 months of life, and weigh benefits and risks of milk consumption.

Ethics and dissemination. The protocol is approved by the Institutional Review Boards of the Ethiopian Public Health Institute (EPHI-IRB-481-2022), Michigan State University (STUDY00007996), and International Food Policy Research Institute (DSGD-23-0102). Written informed consent will be obtained from all participants, who may withdraw from the study at any time. Confidentiality of collected data will be given high priority during each stage of data handling. The study's findings will be disseminated through stakeholder workshops, local and international conferences, journal articles, and technical reports.

Strengths and Limitations of This Study

- This is the first study that assesses how aflatoxin M1 (AFM1) in milk affects child growth in Ethiopia, controlling for socioeconomic, nutritional, and environmental factors.
- This research compares the health risks of AFM1 with the benefits of milk consumption for children.
- The findings will be used to inform feasible, health risk-based policies on AFM1 in milk, not just in Ethiopia, but potentially in other nations worldwide.
- The findings of this study would not allow for causal inference or finding a toxicological mechanism because of the observational design, but would aid in the understanding of the relationship between AFM1 exposure and child growth.

For peer review only

INTRODUCTION

Aflatoxins are secondary metabolites of the fungi *Asperfillus flavus* and *A. parasiticus*, which frequently infect food and feed crops such as maize, groundnuts, oilseeds, and tree nuts (Khlangwiset et al., 2011). Aflatoxin B_1 (AFB_1) is the most toxic form of aflatoxin, and a potent liver carcinogen. Aflatoxin M_1 (AFM_1), a metabolite of AFB_1, is excreted in milk when mammals consume feed contaminated with AFB_1. Infants and young children can become exposed to AFM_1 through dairy animal milk consumption. Although AFM_1 is a far less potent carcinogen than AFB_1, it may cause other health risks. Furthermore, knowledge on the tradeoff between the nutritional benefits of milk vs. risks of AFM_1 exposure in milk is currently sparse.

Globally, milk is a critically important animal source food for infants and young children, providing a wide range of nutrients - protein, carbohydrates, fat, calcium, potassium, and vitamin B12 among others - crucial for growth and development (Dror & Allen, 2011; Iannotti et al., 2013; Miller et al., 2020). Including milk in a children's diets is all the more important when access and affordability of other animal sourced food such as meat and eggs are difficult (Headey & Alderman, 2019). In addition, milk products are economically valuable; as the dairy value chain contributes substantially to livelihoods in semi-urban and rural areas worldwide (Banda et al., 2021; Hegde, 2019; Shackleton et al., 2005).

Conversely, milk can harbor multiple contaminants such as AFM_1. AFB_1, the parent compound of AFM_1, may impair child growth (Wu, 2014) through several pathways such as disruption of insulin-like growth factors (Castelino et al., 2015), intestinal damage (Smith et al., 2012), and immunosuppressive effects that increase infection susceptibility (Saha Turna and Wu, 2022; Saha Turna et al., 2023). Whether AFM_1 could cause similar effects is unknown. This research study aims to fill this knowledge gap by examining the relationship between AFM_1 exposure and growth outcomes in infants and young children in Ethiopia.

Ethiopia is an ideal setting for our proposed work, as it faces important tradeoffs between the benefits of milk production and consumption and AFM_1 risk. Ethiopia has recently adopted a strict standard for AFM_1 in milk, 0.5 μg/L (Ethiopian Standards Agency CES-278), which is often exceeded in raw milk samples from Ethiopia as shown by mean AFM_1 levels ranging between 0.41-0.69 μg/L with a range of 0.028-4.96 μg/L (Saha Turna & Wu, 2021). The action level for AFM_1 in milk in the United States is the same, at 0.5 μg/L (FDA, 2005); while the European Union standard is 0.05 μg/L (EU, 2006). Recent lack of evidence linking AFM_1 to cancer has led to the question of whether the standards set are reasonable (Saha Turna et al. 2022). Moreover, dairy markets are important in Ethiopian livelihoods, particularly in rural areas where dairy is an important source of supplemental income (Dorosh & Minten, 2020). If excessively strict AFM_1 standards are enforced in Ethiopian dairy markets, it could result in both economic losses from discarded milk, and nutritional losses due to negative public perception against milk consumption (Popovic et al., 2017; Saha Turna et al., 2022).

For peer review only - http://bmjopen.bmj.com/site/about/guidelines.xhtml

To assess the relationship between AFM_1 exposure and growth outcomes and the potential benefits and risks of AFM_1 in milk in Ethiopia, we use a prospective longitudinal cohort design. Here, we describe the objectives, methods, and expected outcomes of this study.

OBJECTIVES

Our goal is to determine whether AFM_1 exposure through milk consumption is associated with child growth impairment over a period of 18 months in early childhood; then to compare this risk with the benefits of milk consumption. This evidence will inform policy recommendations for AFM_1 regulation in dairy products in Ethiopia. The specific objectives of this study are to:

1. Determine the association between milk consumption and growth outcomes in children 6-36 months of age.
2. Examine the association between AFM_1 exposure and growth outcomes in children 6-36 months of age who consume milk.
3. Compare the health benefits and risks associated with dairy consumption in relation to child growth outcomes.
4. Develop policy recommendations for regulating AFM_1 and communicating dairy benefits and risks.

METHODS AND ANALYSIS

The study will be carried out in two parts. First, we will conduct formative research with semi-structured interviews of families with young children aged 6-18 months who also own dairy cows, to assess dairy management practices (especially those related to cattle feeds) and dairy production and consumption practices in the Sidama region in Ethiopia. Second, we will use findings to inform the main quantitative arm of the study as outlined in the four objectives above.

Formative Research Study

Limited prior evidence suggests that AFM_1 contamination in milk is widespread in Ethiopia (Admasu et al., 2021; Tadesse & Yilman, 2018). There is also limited evidence on farmers' awareness of AFB_1 contamination in feed and consequently AFM_1 contamination in milk, feed and storage practices used to reduce contamination (if any), and behaviors of children's caregivers in relation to these issues. Hence, we conducted a formative research study to gauge the AFM_1 risk in cow milk in peri-urban and rural study areas with high rates of cattle ownership and variation in market access to feed. Through the formative research, we gained an understanding of cow feeding issues such as feed type, variation, and storage; as well as children's dairy consumption patterns and complementary feeding practices. The findings of the formative research informed the sampling strategy and the questionnaire design of the main study. The results of the formative research will be presented in a different research paper (Anato et al., under review).

For peer review only

Study design and data collection

The target group of the main study is 1000 caregivers and their children aged 6-18 months from urban or peri-urban households that raise dairy cattle. In the initial visit, a baseline survey will be used to collect data such as anthropometric measurements, child's diet, and socio-demographic factors among other data. After the baseline survey, short phone-based interviews will be carried out every 6 months to assess the variability in child's milk consumption and dairy production factors. Our end line visit, mainly to collect data on child's anthropometric measurements, will be carried out 18 months after the initial visit. **Patient and public involvement**: the interview results from the Formative Research described above on public participants in Sidama, Ethiopia, will aid selection of households in the second part of our work.

Study setting

The study will be conducted in two districts, Wondo Genet and Hawassa Zuria of Sidama region in Ethiopia (Fig. 1). These districts are purposely selected due to the common practice of using cow's milk as complementary food for young children, availability of commercial milk, potential for AFB_1 contamination in livestock feeds, and variation in market access to feed.

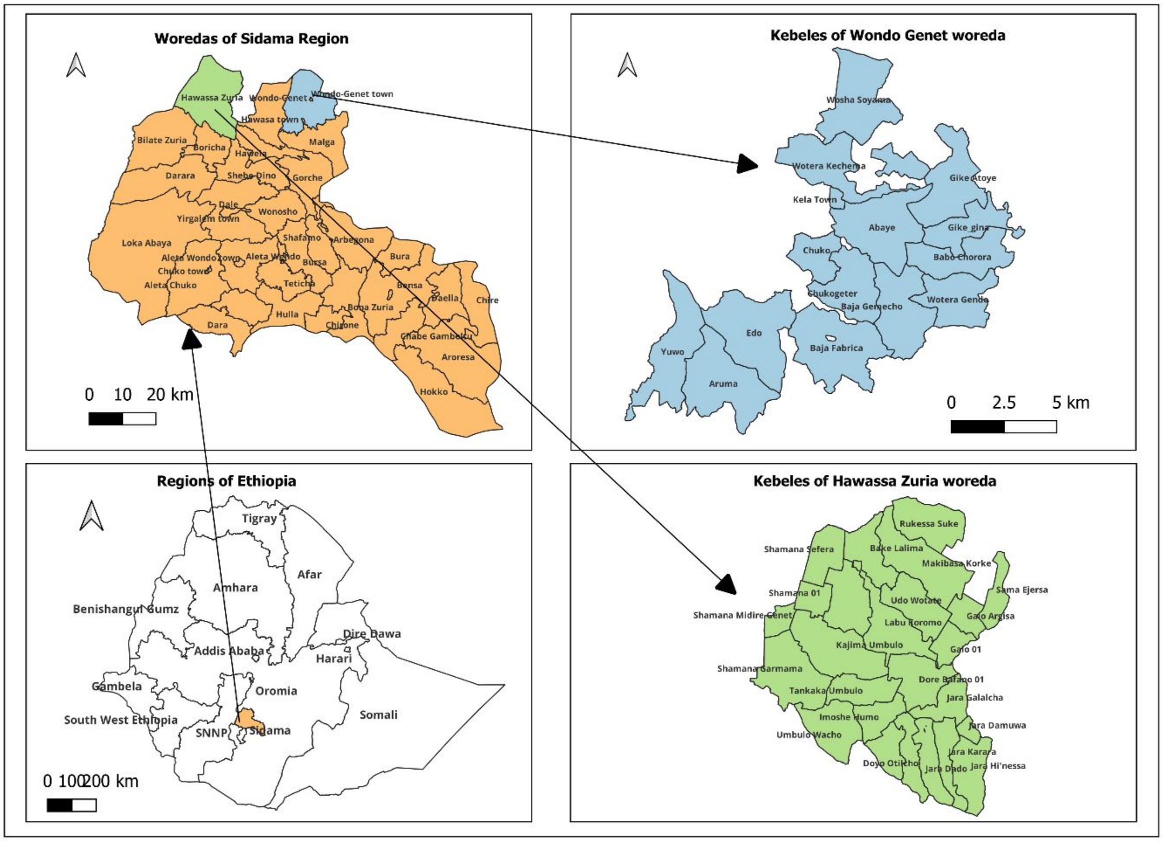

Fig. 1. Maps of the study areas for the proposed work in Sidama, Ethiopia.

Sample size calculations

Using the 2016 Ethiopia Demographic Health Survey (CSA/Ethiopia & ICF, 2017) and focusing on our primary anthropometric outcome variable, we have mean HAZ in children under age 36 months of -1.46 (s.d.=1.56). With the significance level set at 5%, power at 80%, allowing for 10% attrition between survey rounds, and accounting for that not all children consume milk, the study requires approximately 1,000 children to detect a 0.3 standard deviation improvement in child HAZ. This minimum detectable effect size corresponds to the estimated association between household cow ownership and child HAZ (children starting at 6-18 months of age) in a study by Hoddinott et al. (2015) in rural Ethiopia. Since we focus directly on children's dairy consumption rather than cattle ownership, and our exposure variable is continuous rather than dichotomous, the expected magnitude of the association is likely to be larger than the association documented in Hoddinott et al (2015). Thus, we expect this sample size to offer sufficient precision to detect positive and/or negative associations AFM1 exposure and growth outcomes in this population.

Sampling procedures

Our formative research found that feed ingredients and type varied by herd size and season. Based on these findings, we will classify the dairy cow owning households into small herd size (1-3 cattle) and large herd size (4 or more cattle) and randomly select a total of 500 households each from both groups. The two study districts have list of herd sizes to provide the sampling frame. Within these two groups, all households have an equal chance of being selected.

Outcome variables

Anthropometric status indicators are the main outcome variables for this study. Anthropometric measurements – weight, height/length, mid-upper arm circumference (MUAC) and head circumference – will be collected from the children at baseline and endline. Height/length will be measured using height/length boards, with the measurements recorded to the nearest 0.1 cm. Weight will be measured using a digital weighing scale (Seca 874 digital floor scale) and recorded to the nearest 0.1 kg. Head circumference will be measured by using Seca pediatric head circumference measuring band to the nearest 0.1 cm decimal.

Using the WHO 2006 child growth standards, we will calculate length-for-age z-scores (LAZ), weight-for-height z-scores (WLZ), weight-for-age z-scores (WAZ), and head circumference-for-age z-scores (HCZ). The LAZ is the primary variable of interest and as secondary outcomes, we will consider dichotomous variable of stunting (i.e., LAZ below -2 standard deviation (SD)), WLZ and wasting (i.e., WLZ below -2 SD), WAZ and underweight (i.e., WAZ below -2 SD), MUAC, and HCZ.

For peer review only

Explanatory variables

Child's milk consumption and AFM_1 levels in milk are the key explanatory variables in this study. Child's milk consumption will be measured through a one-day quantitative multiple-pass 24-hour recall interview, consisting of four steps designed to enhance respondent's recall accuracy (Gibson & Ferguson, 1999). We will ensure that all days of the week are proportionately represented during the dietary recall, to account for day-of-the-week effects on food intake. We will also collect a second, non-consecutive day 24-hr recall (repeat), within 2 to 10 days of the first recall, from a randomly selected sub-sample of children to account for the day-to-day variability of dietary intake within individuals and for nutrients estimation.

At the baseline and the endline visits, we will collect milk samples (2,000 samples in two rounds). AFM_1 testing in milk, analysis of dilution of milk, nutrients composition, and pathogens testing (including *E. coli, and Campylobacter* spp.,) will be conducted at Ethiopian Public Health Institute (EPHI) laboratory following standard operating practices for handling, processing, and storing samples.

Aflatoxin M1 in milk samples will be detected by using a commercial enzyme-linked immunosorbent assay (ELISA) kit (catering to catalogue no. 961AFLM01C-ULTRA), manufactured by Helica Biosystems Inc. of Santa Ana, CA, USA (Helica, 2021b). The methodology for conducting the test will be based on the manufacturer's guidelines. Standards and samples of 200 µl will be replicated in pre-coated plates. The resulting mixture will be incubated for two hours, followed by washing and addition of conjugates. After the completion of 15 minutes of additional incubation and subsequent washing, enzyme substrate of 100 µl will be added to each well, permitted to incubate for 15 min, and afterwards stopped using 100 µl of stop reaction. Finally, optical density measurements will be taken at 450 nm for each microwell using a microplate reader. Calculation of AFM1 levels achieved by means of a logarithmic standard curve will require an R^2 value of no less than 95%. The average results from duplicate readings will be utilized for the presentation of results (Helica, 2021b).

Control variables

Data on the control variables will be collected during the survey visits and will include characteristics that are likely to be correlated with child growth outcomes and child's milk intake. This includes variables such child's sex, age and diet, parent's education, household wealth index, milk's nutrient content, pathogens in milk and child's maize consumption.

A questionnaire will collect information about child, parent, and household characteristics. Information on children's diet comes from the 24-hour recall survey. We will control for children's diets by calculating caloric intake variables from 8 food groups: 1) Breastmilk, 2) Grains, roots, and tubers, 3) Pulses, nuts, and seeds, 4) Dairy products, 5) Flesh foods (meats, fish, poultry, organ meats), 6) Eggs 7) Vitamin-A rich fruits and vegetables, and 8) other fruits and vegetables (World

For peer review only

Health Organization, 2021). Additionally, we will collect detailed data on the non-standard recipe's ingredients for all mixed dishes that were prepared at home and use standard recipes from the National Food and Nutrition baseline survey dietary dataset to assess the amount of food consumed (Woldeyohannes et al., 2023). The dietary surveys will be administered outside of major Ethiopian Orthodox fasting periods during which dairy household availability and consumption may decline sharply (D'Haene et al., 2020).

One potential source of measurement error in linking food consumption to child growth is within-person variability in consumption. Since linear growth in young children depends on long-term dietary quantity and quality, the 24-hour recall method is imperfect for capturing variability in diets over time (Thorne-Lyman et al., 2014). To address this, we will conduct interim phone surveys every 6 months to ask caregivers the 24-hour milk consumption and weekly recall of the child diet diversity, and assess basic milk production. These higher frequency measurements will be used to assess seasonality, variability, and to adjust for consumption variability in our analysis.

A subsample of 500 livestock feed samples will be collected for testing AFB_1. Detection of AFB_1 in feed samples will be achieved using the Helica Low Matrix Aflatoxin B_1 competitive ELISA kit, manufactured by Helica Biosystems Inc., Santa Ana, CA, listed under Catalogue Number 981BAFL01LM-96 and using procedures outlined by them (Helica, 2021a).

While estimating the child's actual amount of milk intake, the dilution of milk by water, a common practice in Ethiopian livestock sector, is a key concern. The level of dilution from water may not be captured accurately through a survey question, as farmers may underreport such information due to social desirability bias. To address this issue, we will test the milk samples for nitrogen content (approximating protein). The total nitrogen content of the milk will be determined by Kjeldahl method (Lynch & Barbano, 1999).

The milk samples' nutrient composition will be obtained from ongoing analysis of Ethiopian Food Composition tables. However, we will re-analyze randomly selected subsamples to check if their nutrient composition is similar to those reported in the Ethiopian Food Composition table. In brief, proximate composition analysis, moisture content, crude fiber, crude protein, and total ash of milk will be determined; with official method numbers 925.09, 2001.11, 4.5.01, 920.169, and 923.03, respectively (Latimer, 2016). The calcium content of milk will be measured at the EPHI laboratory using an atomic absorption spectrophotometer, following the method no. 985.35 for Ca (Latimer, 2016). The milk samples will be analyzed for pathogens *E. coli* and *Campylobacter* spp. using the general procedures outlined in FDA's Bacteriological Analytical Manual BAM (Feng et al., 2020) and ISO 10272-1:2017 (*for Campylobacter spp.).* We expect that the milk samples found in the study will contain high number of microbial florae in its background and low numbers of *Campylobacter* spp.

Study timeline

For the main study, field activities will commence in November 2023 and all data collection activities will be completed by August 2025. Before the start of the quantitative survey, a one-day stakeholder participatory meeting will be held to inform about the survey goals and seek

For peer review only - http://bmjopen.bmj.com/site/about/guidelines.xhtml

consent to proceed with the study activities. Data management, monitoring and data analysis will be a continuous process throughout the study period. Results dissemination to the stakeholders will be carried out in December 2025.

Data quality assurance

Fifteen days of training for field research assistant and supervisors will be provided. After training on methodological procedures, questionnaires and quality assurance, the questionnaires will be tested in a pilot group and adapted based on the received feedback from the study team. The questionnaires will be translated into the local language (Sidamigna) and back-translated to English to ensure the quality of the translation. Supervisors will receive additional training on teamwork and on monitoring and supervising the data collection process. For laboratory analysis, a quality control chart will be used to ensure the internal and external quality control materials are in the acceptable range.

Data analysis

Empirical approach

Descriptive analysis will focus on computing frequencies and percentages for categorical variables and summary statistics (like means, medians SD, IQR) for summarizing continuous variables. To determine the association between dairy consumption and growth outcomes in children 6-18 months of age at the baseline, we will specify a least squares regression model as:

$$Y_{iv} = \alpha + \beta_M \mathrm{M}_{iv} + \mathrm{X}_{iv}'\delta + \varepsilon_{iv}, \quad (1)$$

where Y_{iv} is the anthropometric outcome for a child i residing in enumeration area v measured at various time points. Variable M_{iv} measures the child's milk intake at various time points. The regression controls for child, parent and household characteristics that are captured in vector X_{iv}. These control variables are observable characteristics that are likely to be correlated both with child's milk intake and his/her growth outcomes. The terms α and ε_{iv} in Equation (1) represent the intercept and error term, respectively. The coefficient β_M quantifies the conditional association between child's milk intake and subsequent growth outcome, measured either as HAZ at the endline or change in HAZ between the baseline and endline surveys. Considering previous evidence from Ethiopia and elsewhere, we expect $\beta_M > 0$.

To determine the association between AFM_1 levels in milk and nutrient density in milk, we will conduct a univariate analysis on each of the macro- and micronutrients and then carryout bivariate tests with AFM_1 levels in the same samples and determine if and how they are associated with each other.

To determine the association between AFM_1 exposure and growth outcomes in children 6-36 months of age who consume dairy, we will test whether the consumption of AFM_1 contaminated milk is associated with lower HAZ (i.e., more growth faltering) controlling for confounders and co-factors, including amount of milk consumed and nutrient content and pathogens in the milk.

We will append Equation (1) with a variable capturing the child's AFM_1 exposure (variable A_{iv}). In this model, we also control for the estimated macro- and micronutrient content and pathogens (vector C'_{iv}) in the milk sample collected at the baseline from the household in which the child resides. We will also control for child's maize consumption (vector H'_{iv}), since maize is also likely to be contaminated by aflatoxins in Ethiopian setting (Chauhan et al., 2016; Getachew et al., 2018; Worku et al., 2019; Yilma et al., 2019). The estimated model can be defined as:

$$Y_{iv} = \alpha + \beta_M M_{iv} + \beta_A A_{iv} + \beta_C C'_{iv} + X'_{iv}\delta + H'_{iv}\vartheta + \varepsilon_{iv}. \quad (2)$$

We expect that $\beta_A < 0$.

To compare the health benefits vs. risks of dairy consumption in child growth outcomes, we will use the regression estimates from Equation (2) to conduct joint hypothesis tests for β_M and β_A, where the null hypothesis is that both of these coefficients are zero, to inform benefit-risk tradeoff of dairy consumption in the context of AFM_1.

Estimation methods

The primary outcome variables in most of our statistical models will be continuous and will therefore typically be estimated using the Ordinary Least Squares (OLS) method. As secondary outcomes we will also examine dichotomous outcomes – such as stunting or wasting – as dependent variables, in which case we will use both linear probability models and Probit models to implement the regression analysis. Homoscedasticity in the residuals will be tested and if rejected, the standard errors will be adjusted for heteroscedasticity following (White, 1980). All statistical analyses will be conducted using Stata, version 17 or higher.

Ethics and dissemination

The study protocol is approved by the Institutional Review Board of the Ethiopian Public Health Institute (protocol no: EPHI-IRB-481-2022), Michigan State University (protocol no: STUDY00007996) and International Food Policy Research Institute (protocol no: DSGD-23-0102). Written informed consent will be obtained from each respondent and participants will be informed that they may withdraw from the study at any time. Confidentiality of all collected data will be given high priority during each stage of data handling. Individual names and personal information of respondents will be kept confidential and data sets will be kept anonymous for analysis. Patient consent for publication is not required.

The study's findings will be disseminated through several communications channels, including stakeholder workshops, various local and international conferences, and technical reports. Additionally, the findings will be shared through research articles and submitted for publication in peer-reviewed journals.

For peer review only

DISCUSSION

The results of this study will inform researchers and policymakers on the impact of AFM_1 in milk on child's nutrition outcomes and will have implications for the transforming dairy value chains in Ethiopia. Ethiopia has recently adopted strict AFM1 standards in milk and other dairy products, which has led to dairy farmers dumping their milk instead of selling it in the market (Saha Turna et al., 2022). An earlier study showed that cancer risk from AFM1 consumption was expected to be extremely low (Saha Turna et al., 2022). However, if AFM1 has other harmful health effects, such as compromising child growth, then the strict regulations could be justified. In this study we can determine whether AFM_1 levels are higher when animals are fed particular feedstuffs, which – if AFM_1 is correlated with poorer child growth outcomes – will help identify which crops should not be included in dairy feed. But if our work finds that AFM_1, independently of livestock feed, causes poor child growth outcomes, then the focus need not just be on reducing AFB1 in dairy feed, but also on technologies that can remove AFM_1 from milk before it is given to children or further processed for other dairy products.

Our work will contribute to rational policymaking and risk communication at local and national levels on whether AFM_1 poses a risk to children's health. It will help policymakers and risk communicators in crafting messages balancing the benefits, risks, and health outcomes associated with dairy consumption. Secondarily, there could be economic benefits to dairy producers if AFM_1 is found to have minimal (or no) adverse child growth outcomes, so that standards and regulations can be set at levels appropriate to the true risk, and so that dairy products do not need to be discarded because of overly stringent regulations (Minten et al., 2020; Miller, 2022).

There are some limitations of our study. Since a randomized control trial in this context is not feasible, we utilize a quasi-experimental approach to understand the risk and benefits of child milk consumption in this context and aim to minimize the risk of bias by controlling for observable confounding factors. We use the 24-hour dietary recall and the limitation of this method is that it only captures food intake in the 24 hours prior to the interview. To address this, we will collect data on the consumption of food groups in the past 7 days and also ask whether the milk quantity consumed in the last 24 hours reflects the typical quantity consumed by the child.

Despite these limitations, the results of our study will inform policy decisions on the appropriate AFM_1 standards for commercial milk and dairy products, on measures to mitigate AFM_1 risks and on improvement of knowledge of AFM_1 risks among dairy consumers, producers, and other dairy value chain actors.

For peer review only

REFERENCES

Admasu, F. T., Melak, A., Demissie, B., Yenew, C., Habtie, M. L., Bekele, T. T., Feyesa, T. O., Chanie, E. S., G/Medhin, M. T., & Malik, T. (2021). Occurrence and associated factors of aflatoxin M1 in raw cow milk in South Gondar Zone, North West Ethiopia, 2020. *Food Science & Nutrition*, *9*(11), 6286–6293.

Banda, L. J., Chiumia, D., Gondwe, T. N., & Gondwe, S. R. (2021). Smallholder dairy farming contributes to household resilience, food, and nutrition security besides income in rural households. *Animal Frontiers*, *11*(2), 41–46. https://doi.org/10.1093/af/vfab009

Castelino, J. M., Routledge, M. N., Wilson, S., Dunne, D. W., Mwatha, J. K., Gachuhi, K., Wild, C. P., & Gong, Y. Y. (2015). Aflatoxin exposure is inversely associated with IGF1 and IGFBP3 levels in vitro and in Kenyan schoolchildren. *Molecular Nutrition & Food Research*, *59*(3), 574–581.

Chauhan, N. M., Washe, A. P., & Minota, T. (2016). Fungal infection and aflatoxin contamination in maize collected from Gedeo zone, Ethiopia. *SpringerPlus*, *5*(1), 753. https://doi.org/10.1186/s40064-016-2485-x

CSA/Ethiopia, C. S. A.-, & ICF. (2017). *Ethiopia Demographic and Health Survey 2016*. CSA and ICF. http://dhsprogram.com/pubs/pdf/FR328/FR328.pdf

D'Haene, E., Vandevelde, S., & Minten, B. (2020). *Fasting, Food, and Farming*. IFPRI-ESSP working paper.

Dorosh, P. A., & Minten, B. (2020). *Ethiopia's agrifood system: Past trends, present challenges, and future scenarios*. International Food Policy Research Institute (IFPRI). https://doi.org/https://doi.org/10.2499/9780896296916

Dror, D. K., & Allen, L. H. (2011). The importance of milk and other animal-source foods for children in low-income countries. *Food and Nutrition Bulletin*, *32*(3), 227–243. https://doi.org/10.1177/156482651103200307

European Union. (2006). Commission Regulation (EC) No 1881/2006 of 19 December 2006 setting maximum levels for certain contaminants in foodstuffs. *Official Journal of the European Union*. https://eur-lex.europa.eu/legal-content/EN/TXT/?uri=CELEX:02006R1881-20230326

Feng, P., Weagant, S., & Jinneman, K. (2020). BAM Chapter 4A: Diarrheagenic Escherichia coli| FDA. *Bacteriological Analytical Manual; Association of Official Analytical Chemists: Washington, DC, USA*.

Getachew, A., Chala, A., Hofgaard, I. S., Brurberg, M. B., Sulyok, M., & Tronsmo, A.-M. (2018). Multimycotoxin and fungal analysis of maize grains from south and southwestern Ethiopia. *Food Additives & Contaminants: Part B*, *11*(1), 64–74.

Gibson, R. S., & Ferguson, E. L. (1999). *An interactive 24-hour recall for assessing the*

For peer review only

adequacy of iron and zinc intakes in developing countries (Issue BOOK). ILSI Press Washington, DC.

Gong, Y. Y., Watson, S., & Routledge, M. N. (2016). Aflatoxin Exposure and Associated Human Health Effects, a Review of Epidemiological Studies. *Food Safety*, *4*(1), 14–27. https://doi.org/10.14252/foodsafetyfscj.2015026

Headey, D. D., & Alderman, H. H. (2019). The Relative Caloric Prices of Healthy and Unhealthy Foods Differ Systematically across Income Levels and Continents. *The Journal of Nutrition*, *149*(11), 2020–2033. https://doi.org/https://doi.org/10.1093/jn/nxz158

Hegde, N. G. (2019). Livestock development for sustainable livelihood of small farmers. *Asian Journal of Research in Animal and Veterinary Sciences*, *3*(2), 1–17.

Helica. (2021a). *Aflatoxin B1 ELISA Assay – Low Matrix 2021*.

Helica. (2021b). *Aflatoxin M1 ULTRA ELISA Quantitative 2021*.

Iannotti, L., Muehlhoff, E., & Mcmahon, D. (2013). Review of milk and dairy programmes affecting nutrition. *Journal of Development Effectiveness*, *5*(1), 82–115.

Khlangwiset, P., Shephard, G. S., & Wu, F. (2011). Aflatoxins and growth impairment: A review. *Critical Reviews in Toxicology*, *41*(9), 740–755. https://doi.org/10.3109/10408444.2011.575766

Latimer, G. W. (Ed.). (2016). *OFFICIAL METHODS OF ANALYSIS OF AOAC INTERNATIONAL - 20TH EDITION, 2016* (20th ed.). AOAC International.

Lynch, J. M., & Barbano, D. M. (1999). Kjeldahl nitrogen analysis as a reference method for protein determination in dairy products. *Journal of AOAC International*, *82*(6), 1389–1398.

Miller, J.D. (2022). Over-regulation of aflatoxin M1 is expensive and harmful in food-insecure countries. *American Journal of Clinical Nutrition* 115:1451-2.

Miller, L., Neupane, S., Joshi, N., & Lohani, M. (2020). MILK Symposium review: Milk consumption is associated with better height and weight in rural Nepali children over 60 months of age and better head circumference in children 24 to 60 months of age. *Journal of Dairy Science*, *103*(11), 9700–9714. https://doi.org/10.3168/jds.2020-18289

Minten, B., Habte, Y., Baye, K., & Tamru, S. (2020). *Food safety, modernization, and food prices: Evidence from milk in Ethiopia* (Vol. 146). Intl Food Policy Res Inst.

Popovic, R., Radovanov, B., & Dunn, J. W. (2017). Food scare crisis: the effect on Serbian dairy market. *International Food and Agribusiness Management Review*, *20*(1), 113–127. https://doi.org/10.22434/IFAMR2015.0051

Saha Turna N, Comstock SS, Gangur V, Chen C, Wu F (2023). Effects of Aflatoxin on the Immune System: Evidence from Human and Animal Research. *Critical Reviews in Food Science and Nutrition*, DOI: 10.1080/10408398.2023.2219336.

Saha Turna, N., Havelaar, A., Adesogan, A., & Wu, F. (2022). Aflatoxin M1 in milk does not

For peer review only

contribute substantially to global liver cancer incidence. *The American Journal of Clinical Nutrition*, *115*(6), 1473–1480.

Saha Turna, N., & Wu, F. (2021). Aflatoxin M1 in milk: A global occurrence, intake, & exposure assessment. *Trends in Food Science & Technology*, *110*, 183–192. https://doi.org/10.1016/J.TIFS.2021.01.093

Saha Turna N, Wu F (2022). Estimation of tolerable daily intake (TDI) for non-carcinogenic effects of aflatoxin. *Risk Analysis* 42:431-8.

Shackleton, C. M., Shackleton, S. E., Netshiluvhi, T. R., & Mathabela, F. R. (2005). The contribution and direct-use value of livestock to rural livelihoods in the Sand River catchment, South Africa. *African Journal of Range & Forage Science*, *22*(2), 127–140. https://doi.org/10.2989/10220110509485870

Smith, L. E., Stoltzfus, R. J., & Prendergast, A. (2012). Food Chain Mycotoxin Exposure, Gut Health, and Impaired Growth: A Conceptual Framework. *Advances in Nutrition*, *3*(4), 526–531. https://doi.org/https://doi.org/10.3945/an.112.002188

Tadesse, G., & Yilman, Z. (2018). Dairy Trade in Ethiopia: Current scenario and way forwardreview. *Journal of Dairy and Veterinary Sciences*, *8*(1), 1–13.

Thorne-Lyman, A., Spiegelman, D., & Fawzi, W. W. (2014). Is the strength of association between indicators of dietary quality and the nutritional status of children being underestimated? *Maternal & Child Nutrition*, *10*(1), 159.

U.S. Food and Drug Administration. (2005). *Compliance Policy Guide. CPG Sec 527.400 Whole Milk, Lowfat Milk, Skim Milk - Aflatoxin M1*. https://www.fda.gov/regulatory-information/search-fda-guidance-documents/cpg-sec-527400-whole-milk-lowfat-milk-skim-milk-aflatoxin-m1

White, H. (1980). A heteroskedasticity-consistent covariance matrix estimator and a direct test for heteroskedasticity. *Econometrica: Journal of the Econometric Society*, 817–838.

Woldeyohannes, M., Girma, M., Petros, A., Hussen, A., Samuel, A., Dinssa, D. A., Challa, F., Laillou, A., Chitekwe, S., & Baye, K. (2023). Ethiopia National Food and Nutrition Survey to inform the Ethiopian National Food and Nutrition Strategy: a study protocol. *BMJ Open*, *13*(4), e067641.

Worku, A. F., Abera, M., Kalsa, K. K., Subramanyam, B., & Habtu, N. G. (2019). Occurrence of mycotoxins in stored maize in Ethiopia. *Ethiopian Journal of Agricultural Sciences*, *29*(2), 31–43.

World Health Organization. (2021). *Indicators for assessing infant and young child feeding practices: definitions and measurement methods*.

Yilma, S., Sadessa, K., & Kebede, D. (2019). Fungal infections and aflatoxin contamination in maize grains collected from west showa and east wallega zones, ethiopia. *International Journal of Current Research Review*, *11*(21), 16–22. http://dx.doi.org/10.31782/IJCRR.2019.11213

For peer review only

Authors' contributions:
FW, DH, KH, MT conceptualized the study, its methodology and drafted the original protocol. MT and AP drafted the first manuscript of the paper. All authors were responsible for reviewing, editing, and approval of final manuscript.

Funding statement: This work was funded in part by the United States Agency for International Development (USAID) Bureau for Food Security under Agreement #AID-OAA-L-15-00003, SUB00003187, as part of Feed the Future Innovation Lab for Livestock Systems. Any opinions, findings, conclusions, or recommendations expressed here are those of the authors alone. The formative research underlying this study was funded by the Bill & Melinda Gates Foundation through the project Advancing Research on Nutrition and Agriculture (AReNA), Phase II (Investment ID: OPP1177007).

Competing interests statement: The authors declare that they have no conflicts of interest.

Word count: 3776 words
World limit: 4000 words (excluding title page, abstract, references, figures and tables)

For peer review only

Dr. Adrian Aldcroft, Editor-in-Chief, *BMJ Open*
January 14, 2024

Dear Dr. Aldcroft: With this letter, we submit our manuscript titled "**Study protocol to assess aflatoxin M1 health risks vs. benefits of dairy consumption in Ethiopian children: An epidemiological trial and risk-benefit analysis**" for your consideration to be published in *BMJ Open.*

Aflatoxin M_1 (AFM_1), a metabolite of aflatoxin B_1 (AFB_1), is excreted in milk when mammals consume feed contaminated with AFB_1. AFB_1, common in maize and nuts, causes liver cancer in humans; and more recently has also been implicated in child stunting, immune dysfunction, and acute liver failure at high doses. The health effects of AFM_1 – if any – are unclear, however. While AFM_1 is a far less potent carcinogen than AFB_1, it may cause other health risks. Relatively speaking, children may be exposed to more AFM_1 than the general population, as they consume the most milk worldwide. At the same time, milk is a highly nutritious food: particularly important for children in regions of the world where animal source foods are scarce. The goal of this study is to determine whether AFM_1 exposure through milk consumption is associated with growth impairment in young Ethiopian children, and to compare this risk with the benefits of milk consumption. The evidence from this study will inform policy recommendations for AFM_1 regulation in dairy products.

This study is longitudinal in design and will be carried out in peri-urban and urban areas of Sidama region in Ethiopia. The target group of this study is 1000 caregivers and their children aged 6-18 months from dairy raising households. Based on the findings of our formative research, we will classify the dairy cow owning households into small herd size (1-3 cattle) and large herd size (4 or more cattle) and randomly select a total of 500 households each from both groups. Anthropometric status indicators are the main outcome variables of this study and AFM_1 levels in milk and milk consumption are key explanatory variables. Control variables include child's age, sex, diet, parent's education, household wealth, milk's nutrient content, pathogens in milk etc.

This study is one of the few studies evaluating the risk of milk consumption through AFM_1 exposure versus benefits of milk consumption among infants and young children. The findings of this study will contribute to rational policymaking and risk communication in regards to AFM_1 levels in milk and dairy products. Hence, this study is suitable for publication in *BMJ Open*.

None of the material pertaining to this study has been published or is under consideration elsewhere, including the internet. All authors have read the manuscript and accept responsibility for its contents.

Our protocol paper is based on a grant proposal that has already been accepted for funding by the United States Agency for International Development (USAID)'s Livestock Systems Innovation Lab. Therefore, I included our proposal online as well. Our grant number is: **#AID-OAA-L-15-00003, SUB00003187**. Therefore, you may consider that our paper does not need to go out to reviewers. If it does: We suggest 2 reviewers who are experts in the field of mycotoxin research:

David Miller, DavidMiller@cunet.carleton.ca
Amare Ayalew, AmareA@africa-union.org

Thank you very much for your consideration of our manuscript. Please do not hesitate to contact me if you have further questions.

For peer review only

Sincerely,

Felicia Wu PhD, John A. Hannah Distinguished Professor
Department of Food Science and Human Nutrition
Michigan State University, 469 Wilson Rd, East Lansing MI USA 48824. Email: fwu@msu.edu

For peer review only

BMJ Open

Study protocol to assess aflatoxin M1 health risks vs. benefits of dairy consumption in Ethiopian children: An epidemiological trial and risk-benefit analysis

Journal:	*BMJ Open*
Manuscript ID	bmjopen-2024-084257.R1
Article Type:	Protocol
Date Submitted by the Author:	14-Mar-2024
Complete List of Authors:	Wu, Felicia; Michigan State University, Department of Food Science and Human Nutrition; Michigan State University, Department of Agricultural, Food, and Resource Economics<br>Headey, Derek ; IFPRI, Poverty, Health and Nutrition Division<br>Hirvonen, Kalle ; International Food Policy Research Institute<br>Pokharel, Ashish; Michigan State University, Department of Agricultural, Food, and Resource Economics<br>Tessema, Masresha; Ethiopian Public Health Institute,
**Primary Subject Heading**:	Epidemiology
Secondary Subject Heading:	Nutrition and metabolism
Keywords:	EPIDEMIOLOGIC STUDIES, Community child health < PAEDIATRICS, PUBLIC HEALTH, NUTRITION & DIETETICS

SCHOLARONE™
Manuscripts

Study protocol to assess aflatoxin M1 health risks vs. benefits of dairy consumption in Ethiopian children: An epidemiological trial and risk-benefit analysis

Felicia Wu[1,2*], Derek Headey[3], Kalle Hirvonen[3], Ashish Pokharel[2], Masresha Tessema[4]

[1] Department of Food Science and Human Nutrition, Michigan State University, East Lansing, MI, USA

[2] Department of Agricultural, Food, and Resource Economics, Michigan State University, East Lansing, MI, USA

[3] International Food Policy Research Institute

[4] Food Science and Nutrition Research Directorate, Ethiopian Public Health Institute, Addis Ababa, Ethiopia

* Correspondence to Dr. Felicia Wu, fwu@msu.edu

For peer review only

ABSTRACT

Introduction. In Sidama, Ethiopia, animal source foods can be difficult to access. Milk has important nutrients for child growth, but carries the risk of aflatoxin M_1 (AFM_1) contamination. AFM_1 is a metabolite of the mycotoxin aflatoxin B_1 (AFB_1) in dairy feed; cows secrete AFM_1 in milk when their feed contains AFB_1 produced by *Aspergillus* fungi in maize, nuts, and oilseeds. It is unknown whether AFM_1 compromises child growth and health.

Methods and analysis. This protocol paper describes our study in Sidama to determine the impact of milk consumption and aflatoxin M_1 on child growth in the first 18 months of life. We will collect baseline and endline data on dairy production, socioeconomic, and nutritional factors of 1000 dairy-owning households with children ages 6-18 months at baseline; and gather samples of milk and dairy feed and child anthropometrics. We will conduct phone interviews every six months to ascertain changes in practices or child health. Dairy feed will be tested for AFB_1; milk for AFM_1, pathogens, and nutrients. Controlling for herd size, socioeconomic, nutritional, and behavioral factors, we will determine the association between child anthropometrics and milk consumption, as well as AFM_1 exposure. We will examine whether AFM_1 exposure affects child growth in the first 18 months of life, and weigh benefits and risks of milk consumption.

Ethics and dissemination. The protocol is approved by the Institutional Review Boards of the Ethiopian Public Health Institute (EPHI-IRB-481-2022), Michigan State University (STUDY00007996), and International Food Policy Research Institute (DSGD-23-0102). Written informed consent will be obtained from all participants, who may withdraw from the study at any time. Confidentiality of collected data will be given high priority during each stage of data handling. The study's findings will be disseminated through stakeholder workshops, local and international conferences, journal articles, and technical reports.

Strengths and Limitations of This Study

- This is the first study that assesses how aflatoxin M_1 (AFM_1) in milk affects child growth in Ethiopia, controlling for socioeconomic, nutritional, and environmental factors.
- This research compares the health risks of AFM_1 with the benefits of milk consumption for children.
- The findings will be used to inform feasible, health risk-based policies on AFM_1 in milk, not just in Ethiopia, but potentially in other nations worldwide.
- The findings of this study would not allow for causal inference or finding a toxicological mechanism because of the observational design, but would aid in the understanding of the relationship between AFM_1 exposure and child growth.

For peer review only

INTRODUCTION

Aflatoxins are secondary metabolites of the fungi *Asperfillus flavus* and *A. parasiticus*, which frequently infect food and feed crops such as maize, groundnuts, oilseeds, and tree nuts (1). Aflatoxin B_1 (AFB_1) is the most toxic form of aflatoxin, and a potent liver carcinogen. Aflatoxin M_1 (AFM_1), a metabolite of AFB_1, is excreted in milk when mammals consume feed contaminated with AFB_1. Infants and young children can become exposed to AFM_1 through dairy animal milk consumption. Although AFM_1 is a far less potent carcinogen than AFB_1, it may cause other health risks. Furthermore, knowledge on the tradeoff between the nutritional benefits of milk vs. risks of AFM_1 exposure in milk is currently sparse.

Globally, milk is a critically important animal source food for infants and young children, providing a wide range of nutrients - protein, carbohydrates, fat, calcium, potassium, and vitamin B12 among others - crucial for growth and development (2–4). Including milk in a children's diets is all the more important when access and affordability of other animal sourced food such as meat and eggs are difficult (5). In addition, milk products are economically valuable; as the dairy value chain contributes substantially to livelihoods in semi-urban and rural areas worldwide (6–8).

Conversely, milk can harbor multiple contaminants such as AFM_1. AFB_1, the parent compound of AFM_1, may impair child growth (9) through several pathways such as disruption of insulin-like growth factors (10), intestinal damage (11), and immunosuppressive effects that increase infection susceptibility (12,13). Whether AFM_1 could cause similar effects is unknown. This research study aims to fill this knowledge gap by examining the relationship between AFM_1 exposure and growth outcomes in infants and young children in Ethiopia.

Ethiopia is an ideal setting for our proposed work, as it faces important tradeoffs between the benefits of milk production and consumption and AFM_1 risk. Ethiopia has recently adopted a strict standard for AFM_1 in milk, 0.5 μg/L (Ethiopian Standards Agency CES-278), which is often exceeded in raw milk samples from Ethiopia as shown by mean AFM_1 levels ranging between 0.41-0.69 μg/L with a range of 0.028-4.96 μg/L (14). The action level for AFM_1 in milk in the United States is the same, at 0.5 μg/L (15) ; while the European Union standard is 0.05 μg/L (16). Recent lack of evidence linking AFM_1 to cancer has led to the question of whether the standards set are reasonable (12). Moreover, dairy markets are important in Ethiopian livelihoods, particularly in rural areas where dairy is an important source of supplemental income (17). If excessively strict AFM_1 standards are enforced in Ethiopian dairy markets, it could result in both economic losses from discarded milk, and nutritional losses due to negative public perception against milk consumption (12,18).

To assess the relationship between AFM_1 exposure and growth outcomes and the potential benefits and risks of AFM_1 in milk in Ethiopia, we use a prospective longitudinal cohort design. Here, we describe the objectives, methods, and expected outcomes of this study.

For peer review only

OBJECTIVES

Our goal is to determine whether AFM_1 exposure through milk consumption is associated with child growth impairment over a period of 18 months in early childhood; then to compare this risk with the benefits of milk consumption. This evidence will inform policy recommendations for AFM_1 regulation in dairy products in Ethiopia. The specific objectives of this study are to:

1. Determine the association between milk consumption and growth outcomes in children 6-36 months of age.
2. Examine the association between AFM_1 exposure and growth outcomes in children 6-36 months of age who consume milk.
3. Compare the health benefits and risks associated with dairy consumption in relation to child growth outcomes.
4. Develop policy recommendations for regulating AFM_1 and communicating dairy benefits and risks.

METHODS AND ANALYSIS

The study will be carried out in two parts. First, we will conduct formative research with semi-structured interviews of families with young children aged 6-18 months who also own dairy cows, to assess dairy management practices (especially those related to cattle feeds) and dairy production and consumption practices in the Sidama region in Ethiopia. Second, we will use findings to inform the main quantitative arm of the study as outlined in the four objectives above.

Formative Research Study

Limited prior evidence suggests that AFM_1 contamination in milk is widespread in Ethiopia (19,20). There is also limited evidence on farmers' awareness of AFB_1 contamination in feed and consequently AFM_1 contamination in milk, feed and storage practices used to reduce contamination (if any), and behaviors of children's caregivers in relation to these issues. Hence, we conducted a formative research study to gauge the AFM_1 risk in cow milk in peri-urban and rural study areas with high rates of cattle ownership and variation in market access to feed. Through the formative research, we gained an understanding of cow feeding issues such as feed type, variation, and storage; as well as children's dairy consumption patterns and complementary feeding practices. The findings of the formative research informed the sampling strategy and the questionnaire design of the main study. The results of the formative research are presented in a different research paper (21).

Study design and data collection

The target group of the main study is 1000 caregivers and their children aged 6-18 months from urban or peri-urban households that raise dairy cattle. In the initial visit, a baseline survey

will be used to collect data such as anthropometric measurements, child's diet, and socio-demographic factors among other data. After the baseline survey, short phone-based interviews will be carried out every 6 months to assess the variability in child's milk consumption and dairy production factors. Our end line visit, mainly to collect data on child's anthropometric measurements, will be carried out 18 months after the initial visit. **Patient and public involvement**: the interview results from the formative research described above on public participants in Sidama, Ethiopia, aided the selection of households in the second part of our work.

Study setting

The study will be conducted in two districts, Wondo Genet and Hawassa Zuria of Sidama region in Ethiopia (Fig. 1). These districts are purposely selected due to the common practice of using cow's milk as complementary food for young children, availability of commercial milk, potential for AFB_1 contamination in livestock feeds, and variation in market access to feed.

Fig. 1. Maps of the study areas for the proposed work in Sidama, Ethiopia.

Sample size calculations

Using the 2016 Ethiopia Demographic Health Survey (22) and focusing on our primary anthropometric outcome variable, we have mean height-for-age z-scores (HAZ) in children under age 36 months of -1.46 (standard deviation (SD) =1.56). With the significance level set at 5%, power at 80%, allowing for 10% attrition between survey rounds, and accounting for that not all children consume milk, the study requires approximately 1,000 children to detect a 0.3 standard deviation improvement in child HAZ. This minimum detectable effect size corresponds to the estimated association between household cow ownership and child HAZ (children starting at 6-18 months of age) in a study by Hoddinott et al. (2015) in rural Ethiopia (23). Since we focus directly on children's dairy consumption rather than cattle ownership, and our exposure variable is continuous rather than dichotomous, the expected magnitude of the association is likely to be larger than the association documented in Hoddinott et al (2015) (23). Thus, we expect this sample size to offer sufficient precision to detect positive and/or negative associations AFM1 exposure and growth outcomes in this population.

Sampling procedures

Our formative research found that feed ingredients and type varied by herd size and season. Based on these findings, we will classify the dairy cow owning households into small herd size (1-3 cattle) and large herd size (4 or more cattle) and randomly select a total of 500 households each from both groups. The two study districts have list of herd sizes to provide the sampling frame. Within these two groups, all households have an equal chance of being selected.

For peer review only

Outcome variables

Anthropometric status indicators are the main outcome variables for this study. Anthropometric measurements – weight, height/length, mid-upper arm circumference (MUAC) and head circumference – will be collected from the children at baseline and endline. Height/length will be measured using height/length boards, with the measurements recorded to the nearest 0.1 cm. Weight will be measured using a digital weighing scale (Seca 874 digital floor scale) and recorded to the nearest 0.1 kg. Head circumference will be measured by using Seca pediatric head circumference measuring band to the nearest 0.1 cm decimal.

Using the WHO 2006 child growth standards, we will calculate height-for-age z-scores (HAZ), weight-for-height z-scores (WLZ), weight-for-age z-scores (WAZ), and head circumference-for-age z-scores (HCZ). The HAZ is the primary variable of interest and as secondary outcomes, we will consider dichotomous variable of stunting (i.e., HAZ below -2 standard deviation (SD)), WLZ and wasting (i.e., WLZ below -2 SD), WAZ and underweight (i.e., WAZ below -2 SD), MUAC, and HCZ.

Explanatory variables

Child's milk consumption and AFM_1 levels in milk are the key explanatory variables in this study. Child's milk consumption will be measured through a one-day quantitative multiple-pass 24-hour recall interview, consisting of four steps designed to enhance respondent's recall accuracy (24). We will ensure that all days of the week are proportionately represented during the dietary recall, to account for day-of-the-week effects on food intake. We will also collect a second, non-consecutive day 24-hr recall (repeat), within 2 to 10 days of the first recall, from a randomly selected sub-sample of children to account for the day-to-day variability of dietary intake within individuals and for nutrients estimation.

At the baseline and the endline visits, we will collect milk samples (2,000 samples in two rounds). AFM_1 testing in milk, analysis of dilution of milk, nutrients composition, and pathogens testing (including *E. coli, and Campylobacter* spp.,) will be conducted at Ethiopian Public Health Institute (EPHI) laboratory following standard operating practices for handling, processing, and storing samples.

Aflatoxin M_1 in milk samples will be detected by using a commercial enzyme-linked immunosorbent assay (ELISA) kit (catering to catalogue no. 961AFLM01C-ULTRA), manufactured by Helica Biosystems Inc. of Santa Ana, CA, USA (25). The methodology for conducting the test will be based on the manufacturer's guidelines. Standards and samples of 200 µl will be replicated in pre-coated plates. The resulting mixture will be incubated for two hours, followed by washing and addition of conjugates. After the completion of 15 minutes of additional incubation and subsequent washing, enzyme substrate of 100 µl will be added to each well, permitted to incubate for 15 min, and afterwards stopped using 100 µl of stop reaction. Finally, optical density measurements will be taken at 450 nm for each microwell using a microplate reader.

For peer review only

Calculation of AFM1 levels achieved by means of a logarithmic standard curve will require an R^2 value of no less than 95%. The average results from duplicate readings will be utilized for the presentation of results (25).

Control variables

Data on the control variables will be collected during the survey visits and will include characteristics that are likely to be correlated with child growth outcomes and child's milk intake. This includes variables such child's sex, age and diet, parent's education, household wealth index, milk's nutrient content, pathogens in milk and child's maize consumption.

A questionnaire will collect information about child, parent, and household characteristics. Information on children's diet comes from the 24-hour recall survey. We will control for children's diets by calculating caloric intake variables from 8 food groups: 1) Breastmilk, 2) Grains, roots, and tubers, 3) Pulses, nuts, and seeds, 4) Dairy products, 5) Flesh foods (meats, fish, poultry, organ meats), 6) Eggs 7) Vitamin-A rich fruits and vegetables, and 8) other fruits and vegetables (26). Additionally, we will collect detailed data on the non-standard recipe's ingredients for all mixed dishes that were prepared at home and use standard recipes from the National Food and Nutrition baseline survey dietary dataset to assess the amount of food consumed (27). The dietary surveys will be administered outside of major Ethiopian Orthodox fasting periods during which dairy household availability and consumption may decline sharply (28).

One potential source of measurement error in linking food consumption to child growth is within-person variability in consumption. Since linear growth in young children depends on long-term dietary quantity and quality, the 24-hour recall method is imperfect for capturing variability in diets over time (29). To address this, we will conduct interim phone surveys every 6 months to ask caregivers the 24-hour milk consumption and weekly recall of the child diet diversity, and assess basic milk production. These higher frequency measurements will be used to assess seasonality, variability, and to adjust for consumption variability in our analysis.

A subsample of 500 livestock feed samples will be collected for testing AFB_1. Detection of AFB_1 in feed samples will be achieved using the Helica Low Matrix Aflatoxin B_1 competitive ELISA kit, manufactured by Helica Biosystems Inc., Santa Ana, CA, listed under Catalogue Number 981BAFL01LM-96 and using procedures outlined by them (30).

While estimating the child's actual amount of milk intake, the dilution of milk by water, a common practice in Ethiopian livestock sector, is a key concern. The level of dilution from water may not be captured accurately through a survey question, as farmers may underreport such information due to social desirability bias. To address this issue, we will test the milk samples for nitrogen content (approximating protein). The total nitrogen content of the milk will be determined by Kjeldahl method (31).

The milk samples' nutrient composition will be obtained from ongoing analysis of Ethiopian Food Composition tables. However, we will re-analyze randomly selected subsamples to check if their nutrient composition is similar to those reported in the Ethiopian Food Composition table. In brief, proximate composition analysis, moisture content, crude fiber, crude

For peer review only

protein, and total ash of milk will be determined; with official method numbers 925.09, 2001.11, 4.5.01, 920.169, and 923.03, respectively (32). The calcium content of milk will be measured at the EPHI laboratory using an atomic absorption spectrophotometer, following the method no. 985.35 for Ca (32). The milk samples will be analyzed for pathogens *E. coli* and *Campylobacter* spp. using the general procedures outlined in FDA's Bacteriological Analytical Manual BAM (33) and ISO 10272-1:2017 (*for Campylobacter spp.). W*e expect that the milk samples found in the study will contain high number of microbial florae in its background and low numbers of *Campylobacter* spp.

Study timeline

For the main study, field activities will commence in April 2024 and all data collection activities will be completed by August 2025. Before the start of the quantitative survey, a one-day stakeholder participatory meeting will be held to inform about the survey goals and seek consent to proceed with the study activities. Data management, monitoring and data analysis will be a continuous process throughout the study period. Results dissemination to the stakeholders will be carried out in December 2025.

Data quality assurance

Fifteen days of training for field research assistant and supervisors will be provided. After training on methodological procedures, questionnaires and quality assurance, the questionnaires will be tested in a pilot group and adapted based on the received feedback from the study team. The questionnaires will be translated into the local language (Sidamigna) and back-translated to English to ensure the quality of the translation. Supervisors will receive additional training on teamwork and on monitoring and supervising the data collection process. For laboratory analysis, a quality control chart will be used to ensure the internal and external quality control materials are in the acceptable range.

Data analysis

Empirical approach

Descriptive analysis will focus on computing frequencies and percentages for categorical variables and summary statistics (like means, medians SD, Inter Quartile Range (IQR)) for summarizing continuous variables. To determine the association between dairy consumption and growth outcomes in children 6-18 months of age at the baseline, we will specify a least squares regression model as:

(1) $$Y_{iv} = \alpha + \beta_M \mathrm{M}_{iv} + \mathrm{X}'_{iv}\delta + \varepsilon_{iv},$$

where Y_{iv} is the anthropometric outcome for a child *i* residing in enumeration area *v* measured at various time points. Variable M_{iv} measures the child's milk intake at various time points. The regression controls for child, parent and household characteristics that are captured in vector X_{iv}.

For peer review only

These control variables are observable characteristics that are likely to be correlated both with child's milk intake and his/her growth outcomes. The terms α and ε_{iv} in Equation (1) represent the intercept and error term, respectively. The coefficient β_M quantifies the conditional association between child's milk intake and subsequent growth outcome, measured either as HAZ at the endline or change in HAZ between the baseline and endline surveys. Considering previous evidence from Ethiopia and elsewhere, we expect $\beta_M > 0$.

To determine the association between AFM_1 levels in milk and nutrient density in milk, we will conduct a univariate analysis on each of the macro- and micronutrients and then carryout bivariate tests with AFM_1 levels in the same samples and determine if and how they are associated with each other.

To determine the association between AFM_1 exposure and growth outcomes in children 6-36 months of age who consume dairy, we will test whether the consumption of AFM_1 contaminated milk is associated with lower HAZ (i.e., more growth faltering) controlling for confounders and co-factors, including amount of milk consumed and nutrient content and pathogens in the milk. We will append Equation (1) with a variable capturing the child's AFM_1 exposure (variable A_{iv}). In this model, we also control for the estimated macro- and micronutrient content and pathogens (vector C'_{iv}) in the milk sample collected at the baseline from the household in which the child resides. We will also control for child's maize consumption (vector H'_{iv}), since maize is also likely to be contaminated by aflatoxins in Ethiopian setting (34–37). The estimated model can be defined as:

$$Y_{iv} = \alpha + \beta_M M_{iv} + \beta_A A_{iv} + \beta_C C'_{iv} + X'_{iv}\delta + H'_{iv}\vartheta + \varepsilon_{iv}. \quad (2)$$

We expect that $\beta_A < 0$.

To compare the health benefits vs. risks of dairy consumption in child growth outcomes, we will use the regression estimates from Equation (2) to conduct joint hypothesis tests for β_M and β_A, where the null hypothesis is that both of these coefficients are zero, to inform benefit-risk tradeoff of dairy consumption in the context of AFM_1.

Estimation methods

The primary outcome variables in most of our statistical models will be continuous and will therefore typically be estimated using the Ordinary Least Squares (OLS) method. As secondary outcomes we will also examine dichotomous outcomes – such as stunting or wasting – as dependent variables, in which case we will use both linear probability models and Probit models to implement the regression analysis. Homoscedasticity in the residuals will be tested and if rejected, the standard errors will be adjusted for heteroscedasticity following (38). All statistical analyses will be conducted using Stata, version 17 or higher.

For peer review only

Ethics and dissemination

The study protocol is approved by the Institutional Review Board of the Ethiopian Public Health Institute (protocol no: EPHI-IRB-481-2022), Michigan State University (protocol no: STUDY00007996) and International Food Policy Research Institute (protocol no: DSGD-23-0102). Written informed consent will be obtained from each respondent and participants will be informed that they may withdraw from the study at any time. Confidentiality of all collected data will be given high priority during each stage of data handling. Individual names and personal information of respondents will be kept confidential and data sets will be kept anonymous for analysis. Patient consent for publication is not required.

The study's findings will be disseminated through several communications channels, including stakeholder workshops, various local and international conferences, and technical reports. Additionally, the findings will be shared through research articles and submitted for publication in peer-reviewed journals.

DISCUSSION

The results of this study will inform researchers and policymakers on the impact of AFM_1 in milk on child's nutrition outcomes and will have implications for the transforming dairy value chains in Ethiopia. Ethiopia has recently adopted strict AFM_1 standards in milk and other dairy products, which has led to dairy farmers dumping their milk instead of selling it in the market (12). An earlier study showed that cancer risk from AFM_1 consumption was expected to be extremely low (12). However, if AFM_1 has other harmful health effects, such as compromising child growth, then the strict regulations could be justified. In this study we can determine whether AFM_1 levels are higher when animals are fed particular feedstuffs, which – if AFM_1 is correlated with poorer child growth outcomes – will help identify which crops should not be included in dairy feed. But if our work finds that AFM_1, independently of livestock feed, causes poor child growth outcomes, then the focus need not just be on reducing AFB1 in dairy feed, but also on technologies that can remove AFM_1 from milk before it is given to children or further processed for other dairy products.

Our work will contribute to rational policymaking and risk communication at local and national levels on whether AFM_1 poses a risk to children's health. It will help policymakers and risk communicators in crafting messages balancing the benefits, risks, and health outcomes associated with dairy consumption. Secondarily, there could be economic benefits to dairy producers if AFM_1 is found to have minimal (or no) adverse child growth outcomes, so that standards and regulations can be set at levels appropriate to the true risk, and so that dairy products do not need to be discarded because of overly stringent regulations (4,39).

There are some limitations of our study. Since a randomized control trial in this context is not feasible, we utilize a quasi-experimental approach to understand the risk and benefits of child milk consumption in this context and aim to minimize the risk of bias by controlling for observable confounding factors. We use the 24-hour dietary recall and the limitation of this method is that it

For peer review only

347 only captures food intake in the 24 hours prior to the interview. To address this, we will collect
348 data on the consumption of food groups in the past 7 days and also ask whether the milk quantity
349 consumed in the last 24 hours reflects the typical quantity consumed by the child.

350 Despite these limitations, the results of our study will inform policy decisions on the
351 appropriate AFM_1 standards for commercial milk and dairy products, on measures to mitigate
352 AFM_1 risks and on improvement of knowledge of AFM_1 risks among dairy consumers, producers,
353 and other dairy value chain actors.

For peer review only

REFERENCES

1. Khlangwiset P, Shephard GS, Wu F. Aflatoxins and growth impairment: A review. Crit Rev Toxicol [Internet]. 2011 Oct 1 [cited 2021 May 31];41(9):740–55. Available from: https://pubmed.ncbi.nlm.nih.gov/21711088/

2. Dror DK, Allen LH. The importance of milk and other animal-source foods for children in low-income countries. Food Nutr Bull [Internet]. 2011 [cited 2021 Jan 13];32(3):227–43. Available from: https://pubmed.ncbi.nlm.nih.gov/22073797/

3. Iannotti L, Muehlhoff E, Mcmahon D. Review of milk and dairy programmes affecting nutrition. J Dev Eff. 2013;5(1):82–115.

4. Miller L, Neupane S, Joshi N, Lohani M. MILK Symposium review: Milk consumption is associated with better height and weight in rural Nepali children over 60 months of age and better head circumference in children 24 to 60 months of age. J Dairy Sci. 2020 Nov 1;103(11):9700–14.

5. Headey DD, Alderman HH. The Relative Caloric Prices of Healthy and Unhealthy Foods Differ Systematically across Income Levels and Continents. J Nutr [Internet]. 2019;149(11):2020–33. Available from: https://www.sciencedirect.com/science/article/pii/S0022316622164886

6. Hegde NG. Livestock development for sustainable livelihood of small farmers. Asian J Res Anim Vet Sci. 2019;3(2):1–17.

7. Banda LJ, Chiumia D, Gondwe TN, Gondwe SR. Smallholder dairy farming contributes to household resilience, food, and nutrition security besides income in rural households. Anim Front [Internet]. 2021 Mar 1;11(2):41–6. Available from: https://doi.org/10.1093/af/vfab009

8. Shackleton CM, Shackleton SE, Netshiluvhi TR, Mathabela FR. The contribution and direct-use value of livestock to rural livelihoods in the Sand River catchment, South Africa. African J Range Forage Sci [Internet]. 2005 Jul 1;22(2):127–40. Available from: https://doi.org/10.2989/10220110509485870

9. Wu F, Groopman JD, Pestka JJ. Public Health Impacts of Foodborne Mycotoxins. Annu Rev Food Sci Technol [Internet]. 2014 Feb 28;5(1):351–72. Available from: https://doi.org/10.1146/annurev-food-030713-092431

10. Castelino JM, Routledge MN, Wilson S, Dunne DW, Mwatha JK, Gachuhi K, et al. Aflatoxin exposure is inversely associated with IGF1 and IGFBP3 levels in vitro and in Kenyan schoolchildren. Mol Nutr Food Res. 2015;59(3):574–81.

11. Smith LE, Stoltzfus RJ, Prendergast A. Food Chain Mycotoxin Exposure, Gut Health, and Impaired Growth: A Conceptual Framework. Adv Nutr [Internet]. 2012;3(4):526–31. Available from: https://www.sciencedirect.com/science/article/pii/S2161831322010286

For peer review only

12. Saha Turna N, Havelaar A, Adesogan A, Wu F. Aflatoxin M1 in milk does not contribute substantially to global liver cancer incidence. Am J Clin Nutr. 2022;115(6):1473–80.

13. Saha Turna N, Comstock SS, Gangur V, Wu F. Effects of aflatoxin on the immune system: Evidence from human and mammalian animal research. Crit Rev Food Sci Nutr [Internet]. :1–19. Available from: https://doi.org/10.1080/10408398.2023.2219336

14. Saha Turna N, Wu F. Aflatoxin M1 in milk: A global occurrence, intake, & exposure assessment. Trends Food Sci Technol. 2021 Apr 1;110:183–92.

15. U.S. Food and Drug Administration. Compliance Policy Guide. CPG Sec 527.400 Whole Milk, Lowfat Milk, Skim Milk - Aflatoxin M1 [Internet]. 2005. Available from: https://www.fda.gov/regulatory-information/search-fda-guidance-documents/cpg-sec-527400-whole-milk-lowfat-milk-skim-milk-aflatoxin-m1

16. European Union. Commission Regulation (EC) No 1881/2006 of 19 December 2006 setting maximum levels for certain contaminants in foodstuffs. Off J Eur Union [Internet]. 2006 Dec 20; Available from: https://eur-lex.europa.eu/legal-content/EN/TXT/?uri=CELEX:02006R1881-20230326

17. Dorosh PA, Minten B. Ethiopia’s agrifood system: Past trends, present challenges, and future scenarios. Washington DC: International Food Policy Research Institute (IFPRI); 2020. 115–146 p.

18. Popovic R, Radovanov B, Dunn JW. Food scare crisis: the effect on Serbian dairy market. Int Food Agribus Manag Rev [Internet]. 2017;20(1):113–27. Available from: https://doi.org/10.22434/IFAMR2015.0051

19. Tadesse G, Yilman Z. Dairy Trade in Ethiopia: Current scenario and way forwardreview. J Dairy Vet Sci. 2018;8(1):1–13.

20. Admasu FT, Melak A, Demissie B, Yenew C, Habtie ML, Bekele TT, et al. Occurrence and associated factors of aflatoxin M1 in raw cow milk in South Gondar Zone, North West Ethiopia, 2020. Food Sci Nutr. 2021;9(11):6286–93.

21. Anato A, Headey D, Hirvonen K, Pokharel A, Tessema M, Wu F, et al. Feed handling practices, aflatoxin awareness and children’s milk consumption in the Sidama region of southern Ethiopia. One Heal [Internet]. 2024;18:100672. Available from: https://www.sciencedirect.com/science/article/pii/S2352771423001921

22. CSA/Ethiopia CSA-, ICF. Ethiopia Demographic and Health Survey 2016 [Internet]. Addis Ababa, Ethiopia: CSA and ICF; 2017. Available from: http://dhsprogram.com/pubs/pdf/FR328/FR328.pdf

23. Hoddinott J, Headey D, Dereje M. Cows, Missing Milk Markets, and Nutrition in Rural Ethiopia. J Dev Stud [Internet]. 2015 Aug 3;51(8):958–75. Available from: https://doi.org/10.1080/00220388.2015.1018903

24. Gibson RS, Ferguson EL. An interactive 24-hour recall for assessing the adequacy of iron and zinc intakes in developing countries. ILSI Press Washington, DC; 1999.

For peer review only

25. Helica. Aflatoxin M1 ULTRA ELISA Quantitative 2021. 2021.

26. World Health Organization. Indicators for assessing infant and young child feeding practices: definitions and measurement methods. 2021;

27. Woldeyohannes M, Girma M, Petros A, Hussen A, Samuel A, Dinssa DA, et al. Ethiopia National Food and Nutrition Survey to inform the Ethiopian National Food and Nutrition Strategy: a study protocol. BMJ Open. 2023;13(4):e067641.

28. D'Haene E, Vandevelde S, Minten B. Fasting, Food, and Farming. IFPRI-ESSP working paper; 2020.

29. Thorne-Lyman A, Spiegelman D, Fawzi WW. Is the strength of association between indicators of dietary quality and the nutritional status of children being underestimated? Matern Child Nutr. 2014;10(1):159.

30. Helica. Aflatoxin B1 ELISA Assay – Low Matrix 2021. 2021.

31. Lynch JM, Barbano DM. Kjeldahl nitrogen analysis as a reference method for protein determination in dairy products. J AOAC Int. 1999;82(6):1389–98.

32. Latimer GW, editor. OFFICIAL METHODS OF ANALYSIS OF AOAC INTERNATIONAL - 20TH EDITION, 2016. 20th ed. AOAC International; 2016.

33. Feng P, Weagant S, Jinneman K. BAM Chapter 4A: Diarrheagenic Escherichia coli| FDA. Bacteriol Anal Manual; Assoc Off Anal Chem Washington, DC, USA. 2020;

34. Getachew A, Chala A, Hofgaard IS, Brurberg MB, Sulyok M, Tronsmo A-M. Multimycotoxin and fungal analysis of maize grains from south and southwestern Ethiopia. Food Addit Contam Part B. 2018;11(1):64–74.

35. Chauhan NM, Washe AP, Minota T. Fungal infection and aflatoxin contamination in maize collected from Gedeo zone, Ethiopia. Springerplus [Internet]. 2016;5(1):753. Available from: https://doi.org/10.1186/s40064-016-2485-x

36. Yilma S, Sadessa K, Kebede D. Fungal infections and aflatoxin contamination in maize grains collected from west showa and east wallega zones, ethiopia. Int J Curr Res Rev [Internet]. 2019;11(21):16–22. Available from: http://dx.doi.org/10.31782/IJCRR.2019.11213

37. Worku AF, Abera M, Kalsa KK, Subramanyam B, Habtu NG. Occurrence of mycotoxins in stored maize in Ethiopia. Ethiop J Agric Sci. 2019;29(2):31–43.

38. White H. A heteroskedasticity-consistent covariance matrix estimator and a direct test for heteroskedasticity. Econom J Econom Soc. 1980;817–38.

39. Minten B, Habte Y, Baye K, Tamru S. Food safety, modernization, and food prices: Evidence from milk in Ethiopia. Vol. 146. Intl Food Policy Res Inst; 2020.

For peer review only

Authors' contributions:

FW, DH, KH, MT conceptualized the study, its methodology and drafted the original protocol. MT and AP drafted the first manuscript of the paper. All authors were responsible for reviewing, editing, and approval of final manuscript.

Funding statement: This work was funded by the Bill & Melinda Gates Foundation Grant # OPP1177007, and by the United States Agency for International Development (USAID) Bureau for Food Security under Agreement #AID-OAA-L-15-00003, SUB00003187, as part of Feed the Future Innovation Lab for Livestock Systems. Any opinions, findings, conclusions, or recommendations expressed here are those of the authors alone. The formative research underlying this study was funded by the Bill & Melinda Gates Foundation through the project Advancing Research on Nutrition and Agriculture (AReNA), Phase II (Investment ID: OPP1177007).

Competing interests statement: The authors declare that they have no conflicts of interest.

Word count: 3776 words

World limit: 4000 words (excluding title page, abstract, references, figures and tables)

For peer review only

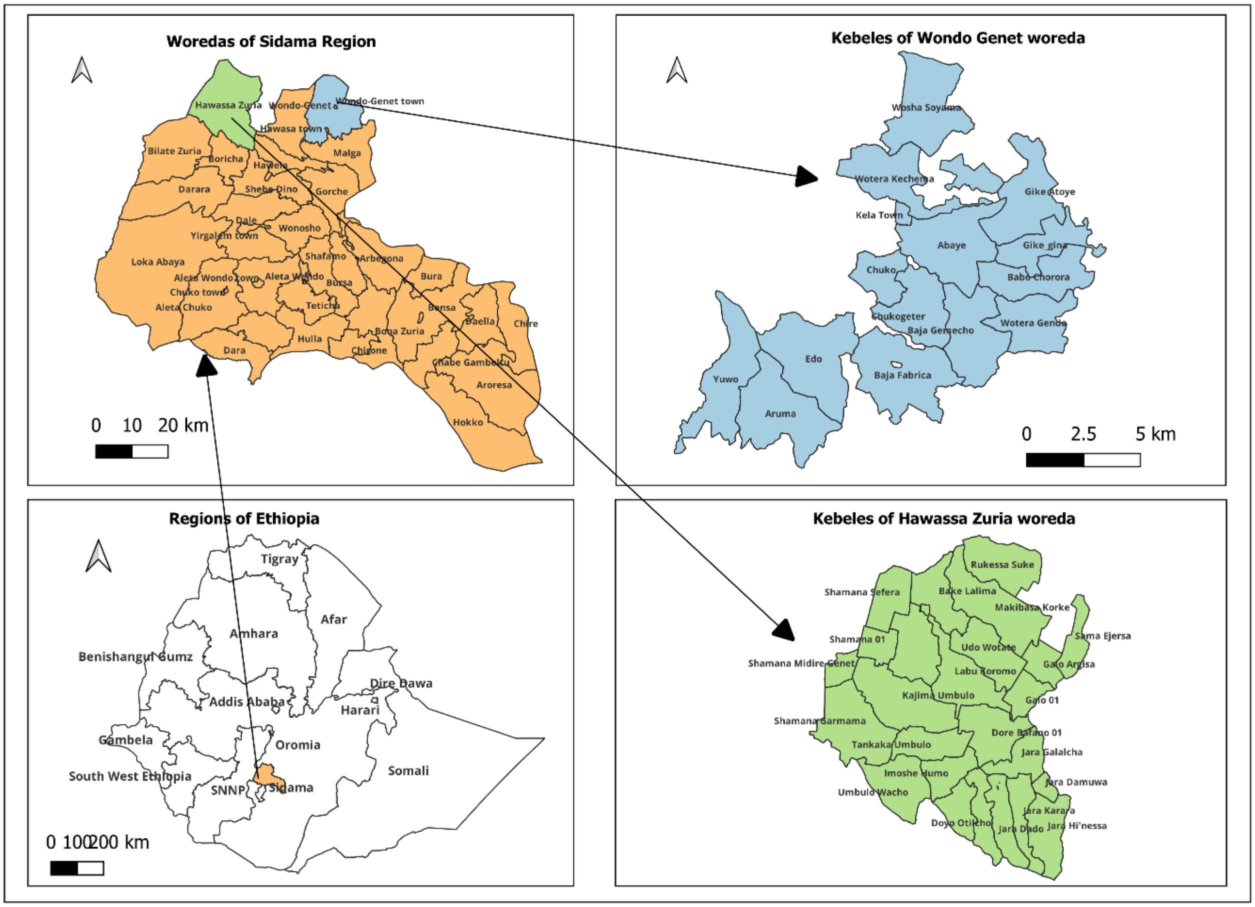

Fig. 1. Maps of the study areas for the proposed work in Sidama, Ethiopia.
