# Supplementary material for: Study protocol to assess aflatoxin M1 health risks versus benefits of dairy consumption in Ethiopian children: an epidemiological trial and risk-benefit analysis
Source: BMJ Open. 2024 Apr 28;14(4):e084257. doi: 10.1136/bmjopen-2024-084257 (PMC11057243; doi:10.1136/bmjopen-2024-084257)
Supplement: Reviewer comments [file bmjopen-2024-084257.reviewer_comments.pdf]

PEER REVIEW HISTORY

BMJ Open publishes all reviews undertaken for accepted manuscripts. Reviewers are asked to complete a checklist review form (**http://bmjopen.bmj.com/site/about/resources/checklist.pdf**) and are provided with free text boxes to elaborate on their assessment. These free text comments are reproduced below.

ARTICLE DETAILS

**TITLE (PROVISIONAL)**	Study protocol to assess aflatoxin M1 health risks vs. benefits of dairy consumption in Ethiopian children: An epidemiological trial and risk-benefit analysis
**AUTHORS**	Wu, Felicia; Headey, Derek; Hirvonen, Kalle; Pokharel, Ashish; Tessema, Masresha

VERSION 1 – REVIEW

**REVIEWER**	Eleni Malissiova<br>University of Thessaly
**REVIEW RETURNED**	14-Feb-2024

**GENERAL COMMENTS**	It is a well structured protocol aiming to answer food security and safety questions
